# *k*-mer-based GWAS in a wheat collection reveals novel and diverse sources of powdery mildew resistance

**DOI:** 10.1186/s13059-025-03645-z

**Published:** 2025-06-18

**Authors:** Benjamin Jaegle, Yoav Voichek, Max Haupt, Alexandros G. Sotiropoulos, Kevin Gauthier, Matthias Heuberger, Esther Jung, Gerhard Herren, Victoria Widrig, Rebecca Leber, Yipu Li, Beate Schierscher, Sarah Serex, Maja Boczkowska, Marta-Puchta Jasińska, Paulina Bolc, Boulos Chalhoub, Nils Stein, Beat Keller, Javier Sánchez-Martín

**Affiliations:** 1 Department of Plant and Microbial Biology, University of Zurich, Zollikerstrasse 107, Zurich, 8008 Switzerland; 2 Gregor Mendel Institute, Austrian Academy of Sciences, Vienna BioCenter, Vienna, 1030 Austria; 3 Leibniz Institute of Plant Genetics and Crop Plant Research (IPK), Corrensstr. 3, Seeland, 06466 Germany; 4 Centre for Crop Health, University of Southern Queensland, Toowoomba, QLD 4350 Australia; 5 Agroscope, Breeding of Crop Plants and Genetic Resources, Plant Breeding, Route de Duillier 60, Nyon, 1260 Switzerland; 6 Department of Microbiology and Genetics, Spanish-Portuguese Institute for Agricultural Research (CIALE), University of Salamanca, Salamanca, 37001 Spain; 7 Plant Breeding and Acclimatization Institute—National Research Institute in Radzikow, Blonie, 05-870 Poland; 8 Crop Plant Genetics, Institute of Agricultural and Nutritional Sciences, Martin-Luther-University of Halle-Wittenberg, Halle (Saale), Germany

## Abstract

Wheat genetic resources hold the diversity required to mitigate agricultural challenges from climate change and reduced inputs. Using DArTseq, we genotype 461 wheat landraces and cultivars and evaluate them for powdery mildew resistance. By developing a *k*-mer-based GWAS approach with fully assembled genomes of *Triticum aestivum* and its progenitors, we uncover 25% more resistance-associated *k*-mers than single-reference methods, outperforming SNP-based GWAS in both loci detection and mapping precision. In total, we detect 34 powdery mildew resistance loci, including 27 potentially novel regions. Our approach underscores the importance of integrating multiple reference genomes to unlock the potential of wheat germplasm.

**Supplementary Information:**

The online version contains supplementary material available at 10.1186/s13059-025-03645-z.

## Background

Wheat production, which accounts for 18% of global calorie intake, is reduced by 20% annually due to pests and diseases [1]. One significant threat is wheat powdery mildew, caused by the obligate biotrophic ascomycete *Blumeria graminis* f. sp*. tritici*. This pathogen can reduce grain yield by 7.6–19.9% [2], leading to annual losses exceeding 4 billion euros worldwide despite the use of agrochemicals, adaptation of agronomic practices, and the deployment of resistance cultivars. Although more than a hundred powdery mildew resistance genes (*Pm*) have been reported, only a few can provide effective resistance in the main wheat-growing areas. For example, from the 16 molecularly cloned *Pm* genes, only a handful are efficient against wheat mildew races in three of the 17 agroecological zones where wheat is grown, leaving vast regions, like Europe and Central Asia, with no effective *Pm* resistance genes [3], forcing breeders to rely on challenging massive evaluation of overall resistance, which remain challenging to implement in breeding programs due to the underlying genetic complexity.

This moderate efficacy of powdery mildew resistance loci in modern varieties is the result of pathogen adaptation as well as a consequence of the narrow genetic base imposed by the bottlenecks of hexaploidization and domestication, exacerbated during the Green Revolution with breeding activities based on few founders. This resulted in genetic erosion and increasing susceptibility and vulnerability to environmental stresses, pests, and diseases, forcing farmers to increasingly rely on pesticides to control wheat diseases, such as powdery mildew. However, chemical control via pesticides is costly, harmful to ecosystems, and increasingly ineffective due to global fungicide resistance [4]. Additionally, the European Commission aims to reduce pesticide usage by 50% by 2030 [5]. In this context, resistance breeding is critical for sustainably controlling pests and pathogens while reducing pesticide dependency.

Plant disease resistance is molecularly diverse and categorized as either race-specific or quantitative resistance (QR). Race-specific resistance provides mostly complete resistance to some pathogen races and only occurs in the presence of a resistance (*R*) gene in the plant, and the corresponding effector-encoding avirulence (*Avr*) gene in the pathogen [6]. In contrast, QR provides partial quantitative resistance at the adult plant stage to all races of a pathogen species, independent of rapidly evolving pathogen effectors [7]. There are only ~ 460 wheat resistance genes genetically defined [8] that are currently being used in breeding. However, based on pan-genome analyses, not representative of all the wheat diversity, up to 7000 resistance genes could be present in the wheat gene pool (Walkowiak et al., 2020). This means that there is a putatively large, unexplored diversity of *Pm* genes stored in wheat gene banks that await being uncovered and used for resistance breeding. In this context, crop wild relatives (CWR) and landraces, genetically diverse populations traditionally cultivated in low-input systems and adapted to different ecoclimatic conditions, represent a valuable genetic resource for improving crops against the occurring climate change.

As an alternative to traditional quantitative trait locus (QTL) mapping, genome-wide association studies (GWAS) offer a faster approach to identifying statistical associations between phenotypic and genetic variations. This method bypasses the need to create segregating populations, significantly reducing the time required. However, there is a notable lack of assessment of the genetic and, in particular, phenotypic diversity of CWR and landraces, limiting their use in breeding [9]. Although genome sequencing costs have dramatically declined, generating reference-quality assemblies of a species like wheat, with its 15-Gb genome, remains both expensive and computationally demanding. Alternatively, various array- and sequencing-based genotyping platforms have emerged to interrogate the genome-wide diversity of wheat genomes. One such method is single-nucleotide polymorphism (SNP)-based arrays, which have proven useful in identifying disease resistance genes in wheat germplasms. However, they typically rely on a single reference genome (or a few accessions), dramatically limiting the SNP set that can be detected [10], and they can only detect SNPs but not the remaining structural variations (SVs), such as insertions, deletions, duplications, copy number variants (CNVs), or translocations that have been shown to underlie relevant traits, such as stress tolerance or disease resistance [11].

To overcome these limitations inherent to SNP-based arrays, alternative genotyping approaches were developed, e.g., Diversity Array Technology sequencing, or DArTseq (http://www.diversityarrays.com/) [9, 12], which produces short sequence fragments by restriction enzyme-mediated genome complexity reduction [13]. This technology selects predominantly low-copy number regions of the genome and by sequencing small regions, typically 200–300 bp, with high coverage, it enables the calling of high-quality SNPs [14]. The resulting SNP information allows interrogating the diversity of hundreds of wheat genotypes at an affordable cost. Such an approach has been successfully applied to wheat in multiple studies [9, 12].

In many model species, only one reference genome is used, and wheat is no exception. Most studies involving wheat genotyping define SNPs or other variations relative to the *Chinese Spring* (CS) reference genome. GWAS based on single reference genomes is restricted to the discovery of genes and variations present in that specific reference genome [15]. However, plants have been shown to have extensive structural variation [16, 17] which cannot be represented by a single reference genome, highlighting the need for more diversity. Further, resistance genes are often part of introgressions from wild relatives or they show presence/absence polymorphism. Consequently, a single reference genome is unlikely to capture all genetic variants [18, 19]. To avoid these two biases, one can use multiple reference genomes to increase the variation detected [20].

Alternatively, an alignment-free approach can be used. This approach differs from the classical SNP-based markers as it directly correlates the presence/absence of small sequences (typically 31 bp), called *k*-mers, with phenotypes. Using *k*-mers as markers allows the detection of almost any type of structural variant, including insertions, deletions, or transpositions in addition to classical SNPs [21]. However, one limitation of alignment-free approaches is the challenge of linking *k*-mers with causal genes.

**Fig. 1:**
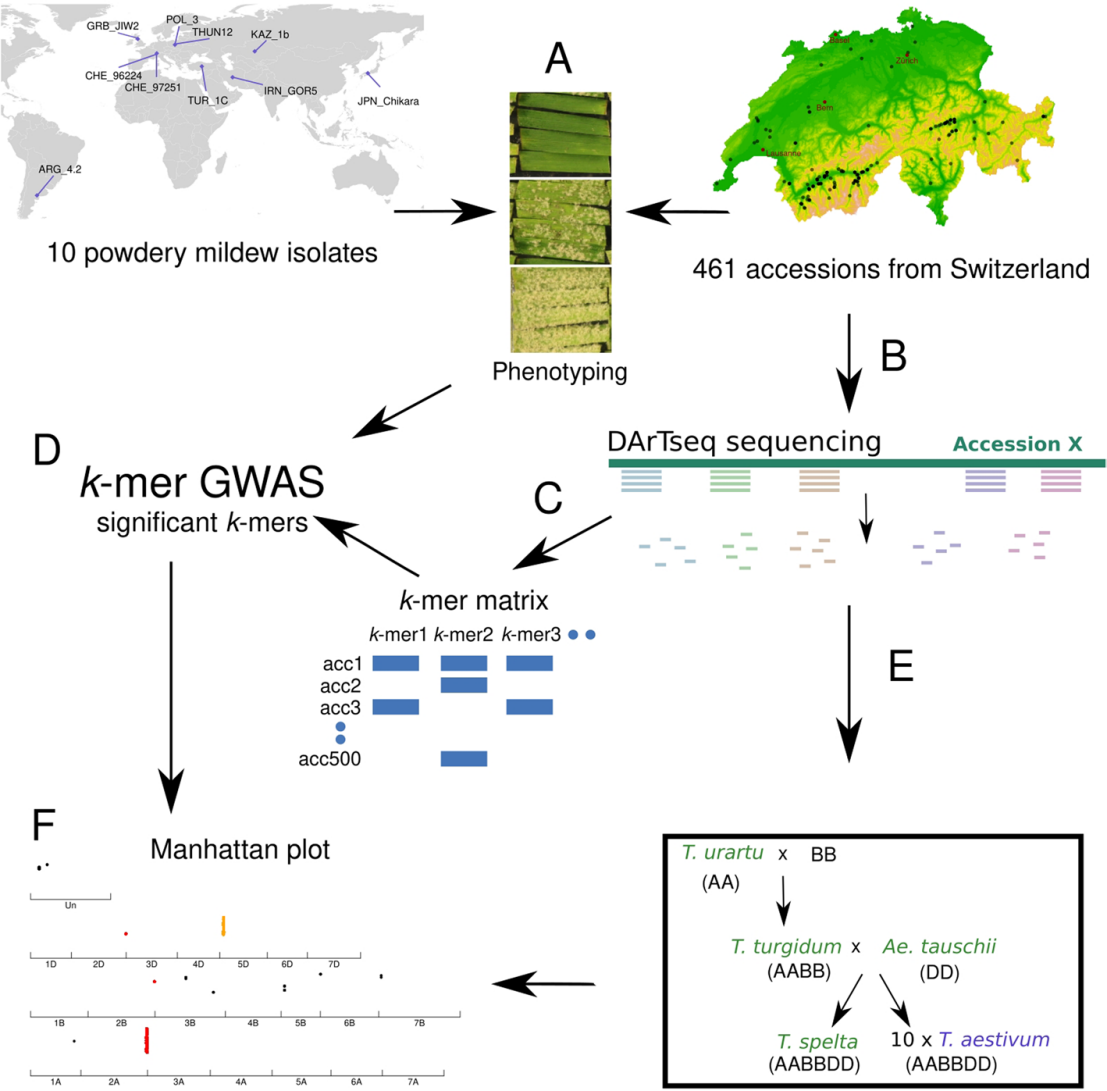
Workflow to identify the genetic basis of resistance in the wheat powdery mildew pathosystem. **A** All accessions from the Swiss wheat collection were phenotyped using 10 *Bgt* isolates from around the globe. **B** All accessions from the collection were sequenced using DArTseq. **C** From the raw reads of the DArTseq, 31-bp *k*-mers were generated, and a presence/absence matrix was used to run GWAS. **D** Using the *k*-mer matrix and the phenotyping data, GWAS was used to find *k*-mers significantly associated with the phenotype. **E** All the DArTseq raw data were mapped to ten *Triticum aestivum* genomes as well as three progenitor genomes and the genome of *T. spelta*. **F** Manhattan plots were generated for each genome of reference. The significant peaks were extracted to select candidate genes

To leverage the untapped genetic diversity of wheat accessions and establish a broadly applicable workflow for plant genomics (Fig. 1), we assembled a diverse collection of 461 Swiss bread wheat accessions (Fig. 1A) and developed a *k*-mer-based GWAS pipeline using multiple wheat reference genomes inspired by work done in flowering plants [21] (Fig. 1B–D). We mapped the raw reads onto ten *Triticum aestivum* reference genomes generated in the 10 + Wheat Genome Project [22], as well as multiple wheat progenitors (Fig. 1E). With this approach, we demonstrated that we could detect a larger diversity of segregating loci involved in powdery mildew resistance that would have been missed using a single reference genome. The association mapping detected the known *Pm* genes *Pm1*, *Pm2*, *Pm60*, or *Pm4b*, but also novel regions associated with powdery mildew resistance in chromosomes 3D, 5D, and 6 A, totaling 34 potential genomic regions of interest spread across all the subgenomes.

Of note, unlike standard GWAS, our method identifies associations with structural variations and sites not present in a single reference genome, highlighting the relevance of landraces and old cultivars stored in genebanks as a source of novel genetic variation important not only for mildew resistance but any other agronomic trait important for adaptation to a changing environment.

## Results

A Swiss wheat collection shows variation in powdery mildew resistance to 10 *Bgt* isolates with different virulence profiles.

**Fig. 2:**
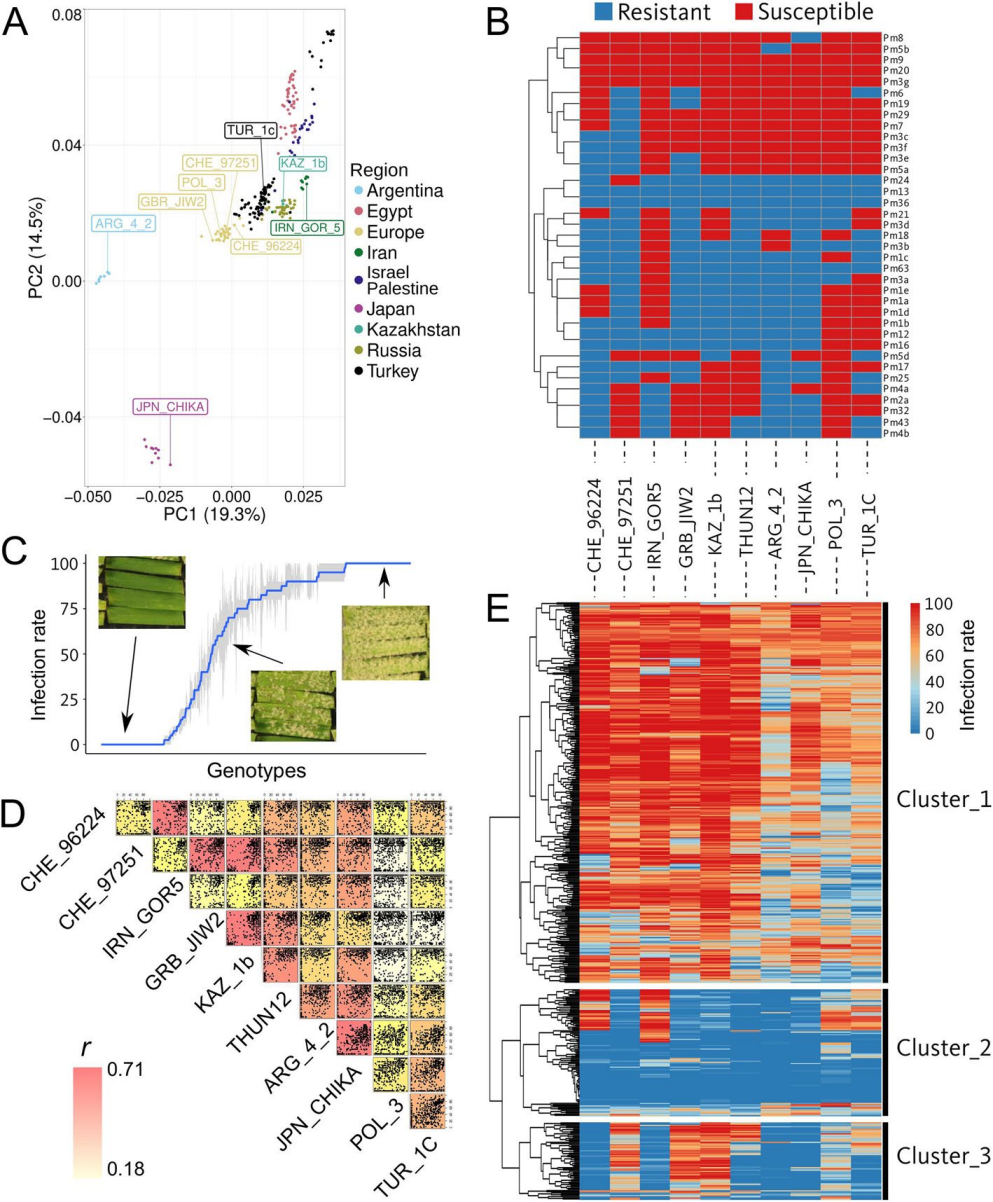
Phenotyping of the Swiss wheat collection with 10 powdery mildew isolates representative of the global genetic diversity of *Blumeria graminis* f. sp. *tritici*. **A** PCA of 400 *Bgt* isolates with 9 of the 10 isolates used in this study highlighted. **B** Avirulence (blue) and virulence (red) pattern of the 10 isolates across 37 Pm-tester lines [24]. **C** Phenotype distribution of the isolate CHE_96224 on the Swiss wheat collection. Pictures represent example phenotypes for fully resistant, partially resistant, and fully susceptible seedling reactions. **D** Correlation plot of the phenotype of all accessions for each isolate. Background color represents the Pearson correlation value. **E** Heatmap representing the phenotype of each accession of the Swiss collection for the ten *Bgt* isolates sorted the same way as **B**. The three main clusters were split

Powdery mildew isolates differ in avirulence gene content and, therefore, the observed reaction to wheat *Pm* resistance genes is isolate-dependent. High-throughput sequencing technologies allow a prediction of effector gene content. As most avirulence genes are still unknown at the molecular level, there are no straightforward tools to select *Bgt* isolates with functional effectors without prior functional validation or extensive phenotyping in tester lines. Therefore, we picked *Bgt* isolates prioritizing diversity, avirulence/virulence spectra, and availability from a global collection [23], ensuring representation from different regions where wheat is cultivated to support a wide search of potential host-resistance components (Fig. 2A).

We selected nine *Blumeria graminis* f. sp. *tritici* isolates from six of the nine populations representing geographical origins detected by [23]. These include two Swiss isolates (CHE_96224 and CHE_97251) from the European cluster, and one from England (GRB_JIW2), Poland (POL_3), Turkey (TUR_1c), Iran (IRN_GOR5), Kazakhstan (KAZ_1b), Argentina (ARG_4_2), and Japan (JPN_CHIKA) each (Fig. 2A). An additional isolate from Poland (THUN12) is a hybrid between wheat and rye mildew, which was not part of the PCA analysis presented in Fig. 2A [23]. The geographic origin of those 10 isolates is shown in Fig. 1A.

To assess the potential of the selected *Bgt* isolates to search for resistance genes, we first determined their avirulence/virulence pattern on 37 *Pm*-tester lines (Fig. 2B). Such tester genotypes have been generated through different crosses, usually involving a wild relative of wheat with a susceptible hexaploid wheat genotype, to ensure the sole presence of a specific *Pm* gene for pathogenicity tests. Across all *Pm*-tester lines, each of the ten isolates revealed a different resistance pattern. Interestingly, only the *Pm13* tester line was resistant to all isolates. In contrast, *Pm9*, *Pm5b*, *Pm20*, and *Pm3g* were susceptible to all ten isolates. Overall, we observed a large variation between the *Pm*-tester lines across the isolates, demonstrating the potential of the chosen *Bgt* isolates for detecting a wide variety of powdery mildew resistance genes (Fig. 2B).

Using the ten chosen *Bgt* isolates, we then phenotyped a collection of Swiss accessions, consisting of 139 and 276 spring and winter accessions, respectively, together with 46 accessions of undefined growth habit (growing in both conditions), totaling 461 accessions. Those accessions include old cultivars and landraces and represent a selection of the diversity held in the Swiss genebank. Mildew resistance phenotyping revealed a large variation in powdery mildew resistance among the collection (Additional file 2: Table S1). Across the 10 isolates, an average of 80 accessions were resistant to individual *Bgt* isolates, while, on average, 354 were susceptible to single *Bgt* isolates (Additional file 2: Table S1). The most virulent *Bgt* isolate, POL_3, had only 32 resistant accessions, while the most avirulent *Bgt* isolate, ARG_4_2, had over 25% (*n* = 146) of the accessions showing resistance. Additionally, 206 accessions were susceptible to all *Bgt* isolates, while only 28 were resistant to all (full data in Additional file 2: Table S1). The latter are of great interest to discover potential broad-spectrum resistance genes.

We developed a Shiny app (https://benjiapp.shinyapps.io/Map_agent_pheno/) to explore the resistance distribution for each *Bgt* isolate. As an example, the resistance distribution is shown for isolate CHE_96224, a very avirulent *Bgt* isolate widely used [25, 26] (Fig. 2C). One hundred thirty-eight accessions showed resistance (≤20% leaf covered) to CHE_96224, whereas 94 accessions were fully susceptible (=100), with 229 accessions showing an intermediate phenotype.

To further explore the commonality between the isolates, we looked at the correlation of their virulence/avirulence patterns across the Swiss collection (Fig. 2D). The highest correlations were observed between JPN_CHIKA and ARG_4_2 (*r* = 0.76) and between CHE_96224 and IRN_GOR5 (*r* = 0.81), with POL_3 being the most distinct *Bgt* isolate with an average correlation value of 0.42 to all other isolates. There was no particular correlation between the geographical origin of the isolate and the virulence/avirulence pattern. For example, the virulence/avirulence patterns of the two isolates from Switzerland, as well as the two from Poland, are poorly correlated with each other compared to the other isolates. Further, we compared the genetic distance (IBS) between isolates and their correlation based on the virulence/avirulence patterns (Additional file 1: Fig. S1), without detecting any significant correlation.

When grouping based on the phenotypic responses to *Bgt* isolates, wheat accessions were grouped into three main clusters. The first and biggest cluster (315 accessions) consists of mostly susceptible accessions to all *Bgt* (Fig. 2E). The second cluster (105 accessions) has mostly accessions resistant to all isolates or accessions susceptible to CHE_96224, IRN_GOR5, POL_3, and TUR_1C *Bgt* isolates. When comparing with the *Pm*-tester lines, such a pattern of accessions being only susceptible to CHE_96224, IRN_GOR5, POL_3, and TUR_1C perfectly matches with the virulence/avirulence patterns of the lines containing *Pm1e*, *a*, and *d*, which suggests that these accessions might contain the *Pm1* locus. The third cluster was formed by 64 accessions that are all resistant to CHE_96224, IRN_GOR5, ARG_4_2, and JPN_CHIKA, while a subset of them are also resistant to THUN12 and TUR_1C. Comparison with the *Pm*-tester lines resistance spectra shows that such a pattern corresponds to *Pm4b* and *Pm2* or *Pm32*-containing lines and indicates that the *Pm2*, *Pm4b*, or *Pm32* genes are likely to be present in the Swiss collection.

The *Pm2* and *Pm4b* race-specific resistance genes are widely present in the Swiss collection.

*Pm4* and *Pm2* are key genes used in breeding programs involved in powdery mildew resistance [27, 28] and have been detected in several wheat populations globally [29, 30]. To investigate whether local wheat hexaploid landraces and old cultivars from the Swiss collection contain these genes as suggested by the phenotyping analysis described above, we tested for the presence of *Pm4b* and *Pm2* using haplotype markers and further sequencing [27, 31]. Out of 461 accessions, 50 and 31 contained *Pm2* and *Pm4b*, respectively, with eight accessions having both genes. Whereas most of the accessions containing *Pm4b* showed the expected resistance to CHE_96224, IRN_GOR5, JPN_CHIKA, THUN12, and TUR_1C [27], few accessions did not match this pattern (Additional file 1: Fig. S2). In the case of expanded resistance, such accessions might contain additional resistance genes, while the lack of resistance is most likely due to the presence of the non-functional *Pm4f* allele [27, 32].

On the other hand, most accessions containing the *Pm2* gene had a resistance spectrum matching the expected pattern of the *Pm2* near isogenic lines (NIL), being resistant against CHE_96224, IRN_GOR5, ARG_4_2, and JPN_CHIKA. The avirulence/virulence pattern of mildew isolates on a *Pm2* NIL perfectly matched the presence/absence of *AvrPm2* in the isolates as determined from whole genome sequences (Additional file 1: Fig. S3). There were a few wheat genotypes without the *Pm2* gene but resistant to *AvrPm2*-containing isolates, which is possibly the result of the presence of other resistance genes. Given the two examples of *Pm2* and *Pm4*, it is very likely that additional sources of resistance are present in the Swiss wheat collection. To discover new genes involved in powdery mildew resistance, we set out to perform GWAS analyses.

### Interrogating genetic diversity in the Swiss wheat collection using DArTseq genotyping

All accessions from the Swiss wheat collection were genotyped using DArTseq. This genotyping by sequencing (GBS) method aims to reduce the proportion of repetitive sequences, keeping genome coverage more homogenous [13]. To assess this, we analyzed the distribution of the sequencing reads across the Chinese Spring IWGSC_2.1 reference genome (CS, [33]). We observe that at the population level 40% of the genes contain at least one read in their coding regions and when including ±10 kb there are 72% of the genes with at least one read in the region (Additional file 1: Fig. S4). While this does not directly reflect the robustness of locus discovery, it illustrates the genomic breadth captured across genic and intergenic regions. Additionally, the distribution of reads was similar between homologous chromosomes with 1,491,774, 1,605,237, and 1,350,862 reads mapping to the subgenomes A, B, and D, respectively (Additional file 1: Fig. S5). As in all GBS methods, DArTseq covers a small part of the genome and produced on average 1.4M reads per sample (Supplemental Table 1, sheet 3). Additionally, we observe that on average, 2.3% of the reads per accession did not map to the CS genome. AG-392 had the fewest mapped reads, with 8.8% not mapping to the CS genome.

The collection was also genotyped with the 12k Illumina Infinium 15 K wheat SNP array (TraitGenetics GmbH, Gatersleben, Germany). After filtering, 11,252 SNPs were kept (see “Methods”). In addition to the SNP array, a matrix of 10,068 SNPs was generated by mapping the DArTseq reads to the CS genome. When comparing the number of SNPs per chromosome for each matrix, the D subgenome showed the expected lower number of SNPs (Additional file 1: Fig. S6). This reduced diversity is due to a strong bottleneck in the formation of hexaploid wheat [34]. Importantly, while the SNP matrix derived from DArTseq data, containing 10,068 SNPs, tagged 2789 genes, using *k*-mers, ~ 85,000 genes (72%) with ± 10 kb window are tagged (Additional file 1: Fig. S4). In addition to the two SNP matrices, we generated a presence/absence matrix of 176 million unique *k*-mers of length 31 bp. We then used these two SNP-based matrices (DArTseq, the SNP-chip) and the *k*-mer matrix—to perform GWAS. Using the SNPs, we also performed a population structure analysis of the Swiss collection using ADMIXTURE. Through the different criteria presented in Additional file 1: Fig. S7, we do not detect multiple populations in our dataset.

*K*-mer-based association mapping outperforms GWAS based on SNP matrices.

**Fig. 3:**
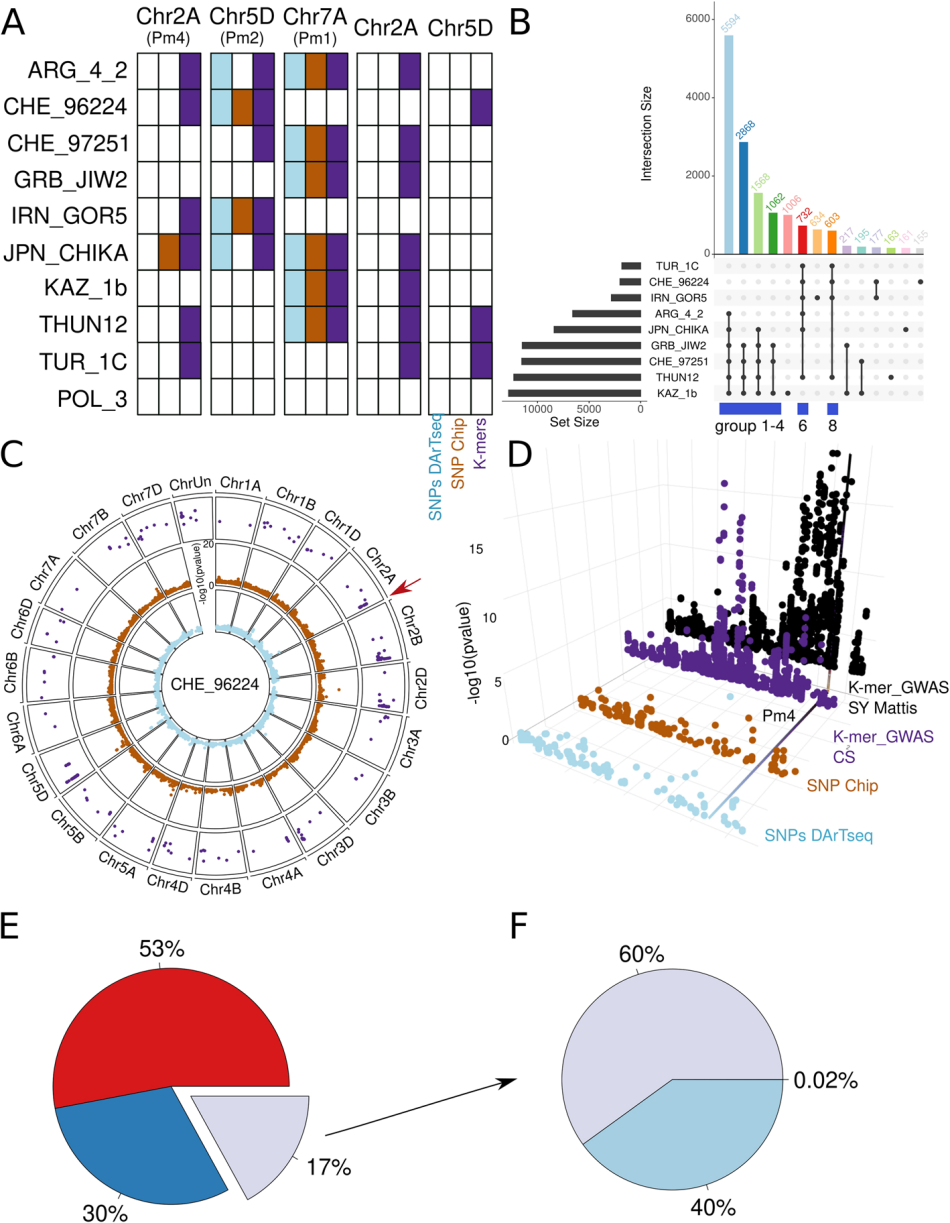
*k*-mer GWAS results. **A** Summary of the GWAS results based on CS for *Pm1*, *Pm2*, and *Pm4* for all three matrices for all the isolates, as well as two regions only found using *k*-mers. The colors blue, brown, and purple respectively represent the SNP-Chip, the SNPs generated from the DArTseq data, and the *k-*mers. Filled squares represent the presence of an associated region.** B** UpSet plot for all *k*-mers significantly associated with resistance using the resistance phenotype of each of the 10 isolates. The biggest set of common significantly associated *k*-mers (5594) was found between the isolates ARG_4_2 and JPN_CHIKA, GRB_JIW2, CHE_97251, THUN12, and KAZ_1b. The first 14 groups are colored. The entire data set can be explored in the Shiny app at https://benjiapp.shinyapps.io/Manhattan_plot/. **C** Circular plot representing Manhattan plots comparing three matrices used for the GWAS of *Bgt* isolate CHE_96224. The inner circle in blue represents the SNP matrix generated using DArTseq, and the middle circle in orange represents the matrix from the SNP-chip. The outer circle represents the *k*-mer-GWAS where only *k*-mers significantly associated with resistance are displayed. The y-axis is the same for all three plots. **D** 3D dot plot of the different Manhattan plots for the region of *Pm4* (red arrow on **C**). The color legends are as in **A**. The *k*-mer-GWAS SY Mattis represents the *k*-mer-GWAS using the SY Mattis genomes as a reference. The line is at the position of the *Pm4* gene in the SY Mattis genome. **E** Proportion of *k*-mers mapping to all the *Triticum aestivum* genomes (red), some of the genomes (blue), and none of the genomes (gray).** F** Proportion of reads that do not map to any of the *T. aestivum* genomes, but map to genomes of wheat progenitors or relatives. The other 40% do not map to any (light blue) and only 0.02% map to all four genomes

To perform a comparative analysis of the performance of GWAS when using the different genotyping methods, we set up a pipeline allowing the use of the three matrices and the mildew resistance as phenotype. The GWAS results using the two SNP matrices showed very similar results (Additional file 1: Figs. S8 and S9). With both SNP matrices, for the *Bgt* isolate POL_3, no significant regions were detected. Significance when using the SNP matrix was set based on the Bonferroni threshold, with any SNP above this threshold considered significantly associated with the phenotype. We also evaluated the fit of the GWAS model to our data and generated QQ plots (Additional file 1: Fig. S10) for all the isolates using the SNPs chip dataset. In all cases, the distribution of *p* values fits well the theoretical distribution of the linear mixed model. Combining the GWAS results from all the other isolates, we detected 241 and 53 significantly associated SNPs from the DArTseq and the SNP-chip, respectively. They were embedded in seven genomic regions, with two regions standing out above the rest: one located at the beginning of chromosome 5D for resistance to CHE_96224 and IRN_GOR5, and one at the end of chromosome 7 A for resistance to ARG_4_2, CHE_97251, GRB_JIW2, JPN_CHIKA, KAZ_1b, and THUN12 (Fig. 3A). Those two regions are known to contain *Pm* genes: *Pm2*, located at the beginning of chromosome 5D around 43.4 Mb [35], and *Pm1* and *Pm60* are located at the end of chromosome 7 A [36, 37]. We observed that the corresponding regions on homoeologous chromosomes also showed significantly associated SNPs. This is most likely due to the homology of sequences between the chromosomes and/or linkage disequilibrium. Based on the phenotyping clustering as well as the comparison with *Pm*-tester lines, *Pm1/Pm60* and *Pm2* were expected to be present in the Swiss wheat collection. Surprisingly, we did not detect *Pm4* using the SNP matrices.

To test whether the output of the GWAS analysis could be improved by using a reference-free approach, we adapted a previously published *k*-mer GWAS approach [21]. The GWAS using *k*-mers as genotype yielded 16,895 *k*-mers significantly associated with resistance, with an average of 5876 *k*-mers per isolate. *k-*mers significantly associated with resistance were defined as those exceeding the − log10 threshold for the 5% family-wise error rate [21]. Isolate KAZ_1b had the highest number of significant *k*-mers (12,806) associated with resistance, and TUR_1C, the lowest number (1819) (Fig. 3B). Similar to the SNPs, no *k*-mers significantly associated with resistance were detected for the POL_3 isolate (summary in Additional file 2: Table S3). Using the SNP matrices, the classical outputs are lists of significant SNPs; for *k*-mers, the output is a list of significant *k*-mers, of 31 bp in length. To position these *k*-mers within a genome, several steps are needed: after retrieving the reads containing the *k*-mers, these reads are aligned to the genome and then the *k*-mer *p* value can be linked to a genome position (see Fig. 1 for the pipeline).

*k*-mer GWAS identified the same resistance-associated regions as the two SNP matrices, plus additional novel associations. For example, we detected an associated region on chromosome 2A that corresponds to the location of the *Pm4* gene (Fig. 3C, D). Based on the resistance pattern analysis of the phenotypic data and the haplotype analysis (Additional file 1: Fig. S2), this gene is present in the Swiss wheat collection. To better understand why such a region is only detected using *k*-mers, we zoomed into the *Pm4* region and plotted the results of the three approaches together (Fig. 3D). This revealed that the higher resolution of the *k*-mer GWAS was due to the higher number of markers in the region: there are 165, 141, and 1627 markers for the DArTseq, the SNP-chip, and the *k*-mer-GWAS, respectively.

Using *k*-mer GWAS approach, we detected additional associated regions 2 A and 5D (Fig. 3A). In total, using Chinese Spring as reference, we identified 21 associated regions that do not overlap with known *Pm* genes (coordinates summarized in Additional file 2: Tables S8 and S9). Manhattan plots containing all the significantly associated regions for all the isolates are presented in Additional file 1: Fig. S12. These results demonstrate that *k*-mer-based GWAS outperforms approaches based on SNPs, and the comparative analysis highlights that the increased number of markers is mostly the reason for this improvement.

Multiple reference genomes improve the positions of *k*-mers significantly associated with resistance and refine genomic region definitions.

Of the 16,895 *k*-mers significantly associated with resistance, 68% (11,570) were mapped to the CS reference genome. In contrast, while only 2.3% of the overall reads did not map to the CS genome, a much larger proportion, i.e., 32% of the *k*-mers significantly associated with resistance, did not map to the CS genome. This suggests a significant enrichment of non-mapping *k*-mers among those identified as significantly associated with resistance, compared to the general read mapping. Such a difference is most likely caused by introgressions and/or presence/absence polymorphisms that are frequently the origin of resistance to powdery mildew [38]. If such an introgressed region is not present in CS, reads containing significantly associated *k*-mers would not map. To investigate the possible origin of the non-mapped *k*-mers, we used 10 additional *Triticum aestivum* genomes [22] as reference genomes. Using this set of genomes, we found that 53% (8969) of the *k*-mers significantly associated with resistance were mapped in all genomes (Fig. 3E). We considered a *k*-mer mapped when at least one of the reads containing such *k*-mers mapped to one or more genomes. A further 30% (5003) were mapped in some but not all of the genomes, and the remaining 17% (2923) did not map to any of the 10 *Triticum aestivum* genomes (Fig. 3E). Thus, by adding the 10 genomes as references, we could map 83% of the *k*-mers, compared to 68% when using only CS as a reference. Some of the *k*-mers that do not map to CS map to new regions of interest in other reference genomes. Besides, some *k*-mers also map to regions that have already been detected using CS, but reduce the width and increase the precision of the peak. Taking *Pm4* as an example, we found that when using SY Mattis as a reference genome, the associated region perfectly overlaps with the position of the annotated *Pm4* gene, which is not the case for CS (Fig. 3D). The *Pm4* gene is known to originate from an introgression from tetraploid wheat [27], and blast analysis shows that the gene is present in SY Mattis and absent in CS. This explains the higher precision when using SY Mattis as a reference genome. Using the *k*-mer approach, we pinpointed narrower peaks, allowing us to define more precisely genomic regions harboring genetic loci of interest (Fig. 3D).

Progenitor genomes allow mapping an additional 8% of the significant *k*-mers.

Many introgressions derived from wild relatives in bread wheat have been described to contain resistance genes [39, 40]. Therefore, to investigate the possible origin of the 17% non-mapped reads, we selected a set of genomes representing close relatives of bread wheat as well as ancestors/progenitors of hexaploid wheat, including *Aegilops tauschii*, *Triticum turgidum*, *Triticum urartu*, and *Triticum spelta* (Fig. 1). By implementing this step, we mapped an additional 60% (1742) of the 2923 non-mapped *k*-mers (Fig. 3E, F). Combining all reference genomes, we could unambiguously assign a physical position to approximately 93% of the *k*-mers, which is 25% more of the resistance-associated *k*-mers compared to using only CS as a reference genome. In summary, our analysis demonstrates the advantage of using multiple reference genomes: starting from the same set of raw genotypic data, an additional 25% of all significant *k*-mers were mapped, resulting in novel associations and improved resolution of associated genomic regions.

*k*-mer GWAS allows to detect multiple known *Pm* genes present in the Swiss wheat collection.

To identify resistance genes acting against multiple *Bgt* isolates, we analyzed resistance-associated *k*-mers shared among multiple isolates (Fig. 3B). Due to the complexity of displaying association data from multiple isolates across different genomes, we developed a Shiny app, accessible at https://benjiapp.shinyapps.io/Manhattan_plot/. To generate a Manhattan plot, users must follow the following steps: (i) select a reference genome, (ii) choose an isolate, and (iii) specify the chromosome (s) to display. While only one genome can be visualized at a time, multiple isolates can be compared simultaneously by stacking multiple plots. For a more detailed view of an associated region, users can zoom in by specifying the coordinates of a region of interest. An annotated screenshot is shown in Additional file 1: Fig. S13.

The largest group of common resistance-associated *k*-mers (*n* = 5594) is shared among the isolates ARG_4_2, JPN_CHIKA, GRB_JIW2, CHE_97251, THUN12, and KAZ_1b. These *k*-mers predominantly cluster at the end of chromosome 7 A, a region known to harbor the *Pm1* and *Pm60* resistance genes, and is characterized by suppressed recombination [37]. This clustering aligns perfectly with the phenotype of the *Pm1d/a/e* tester lines, which exhibits resistance to the abovementioned isolates and susceptibility to the rest (Fig. 2B). Furthermore, *k*-mers from this group also tag other loci of interest, such as on chromosomes 2B and 6B. Extending this to all the reference genomes we found, for instance, that an associated region at the beginning of chromosome 3D was only detected when using Renan, Lancer, and CS as reference genomes. Out of the 34 main regions detected across the 10 reference genomes and the 10 isolates, only 15 are shared in all reference genomes. Such comparative analysis across multiple genomes highlights the limitations of using a single genome reference, as many significant regions would be missed.

The first four groups (groups 1–4) in Fig. 3B represent the largest groups of common *k*-mers and are formed by a similar set of isolates with minimal variation. Group 5 comprises *k*-mers private to KAZ_1b, while groups 6 and 8 contrast with the first four groups as they contain *k*-mers found significant for isolates TUR_1C, CHE_96224, and IRN_GOR5. THUN12 isolate is the only member common among all these groups. Group 7 includes significant *k*-mers solely from IRN_GOR5 (Fig. 3B). This pattern correlates well with the correlation matrix between the phenotypes shown in Fig. 2C.

Interestingly, we observed two distinct, non-overlapping groups of isolates sharing common *k*-mers. One group includes *Bgt* isolates GRB_JIW2, CHE_97251, and KAZ_1b, while the other group comprises *Bgt* isolates TURC_1C, CHE_96224, and IRN_GOR5. Comparing these data with the pattern of *Pm*-tester lines, we found that a combination of multiple *Pm* genes could explain this distribution. In particular, *Pm2a* and *Pm4a* confer resistance to TURC_1C, CHE_96224, and IRN_GOR5, while showing susceptibility to the other isolates, except for JPN_CHIKA. Conversely, various *Pm1* alleles exhibited resistance to GRB_JIW2, CHE_97251, and KAZ_1b. Examining the mapping of the significantly associated *k-*mers from different groups revealed a clear correlation with the expected *Pm* genes. Groups 1–4 predominantly map to the end of chromosome 7, around the *Pm1* and *Pm60* region. For example, for the isolate CHE_97251, out of the 1457 significantly associated *k*-mers mapping to the CS genome, 888 (61%) are located at the end of Chr7A-B-D that contain the resistance genes *Pm1* and *Pm60*. *k*-mers from groups 6 and 8 almost exclusively tag the *Pm2* and *Pm4* regions. For the isolate CHE_96224, out of the 285 significantly associated *k*-mers mapping to the SY Mattis genome, 250 (87%) map to chromosome 2 A in the *Pm4* region. While we detected associated regions for *Pm2 *and* Pm1/Pm60* in all the ten reference genomes, the *Pm4* region is not detected in the Lancer genome.

**Fig. 4:**
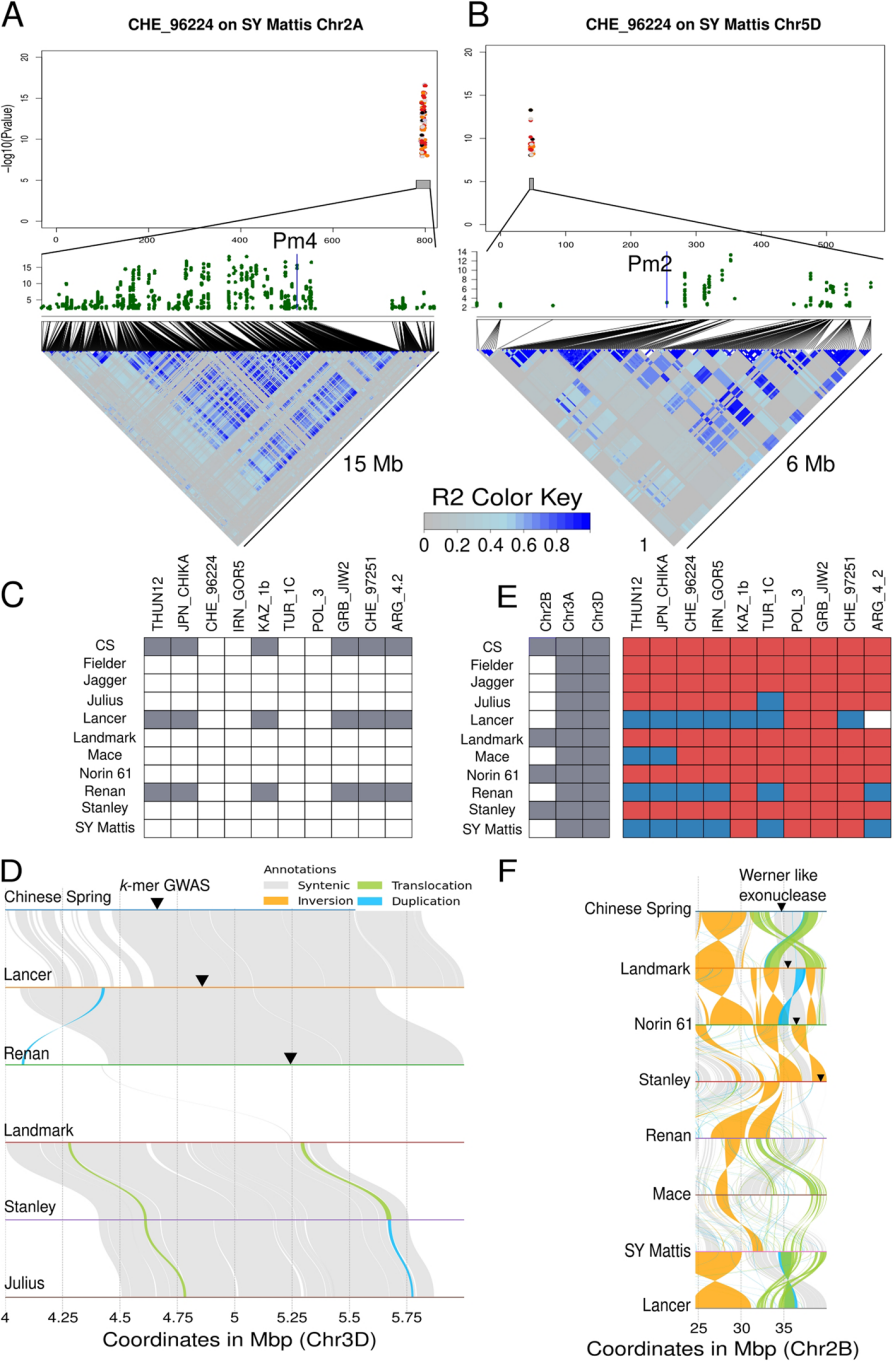
Identification of known *Pm* genes and new candidate genes for powdery mildew resistance. Manhattan plot for the *Bgt* isolate CHE_96224 and the chromosomes 2 A (**A**) and 5D (**B**) of the SY Mattis genome. The zoom-in of each of the regions of interest also shows the position of two of the *Pm* genes known to confer powdery mildew resistance as well as the LD pattern of the region. **C** Table summarizing the presence of the three copies of the Werner-like candidate genes across 11 assembled wheat genomes (gray part). Phenotypes of the same 11 genotypes for the 9 [10] isolates used in this study. Blue represents resistance, red susceptibility, and white is missing data. **D** Alignment of the genomic regions (15 Mb) on chromosome 2B containing the Werner-like exonuclease gene candidate for genomes containing the candidate gene (CS, Landmark, Norin 61, and Stanley), as well as other genomes not containing it (Renan, Mace, SY Mattis, and Lancer). **E** Presence/absence pattern of the associated region at the beginning of Chr3D for 11 genomes and for each of the isolates used in this study. **F** Alignment of the genomic region around the candidate gene at the beginning of chromosome 3D (4 to 6 Mb). The position of the GWAS peak, as well as the best gene candidates, is marked with a triangle. A longer alignment of the 12 first Mb of chromosome 3D is in Supplemental Fig. 16

Such grouping together with the mapping to multiple genomes shows that we can detect clear and strong signals from specific *Pm* genes. Zooming into the region of *Pm4* and *Pm2* using SY Mattis as reference genome shows that those signals are directly on top of the position of the genes (Fig. 4A and B).

New candidate *Pm* genes identified in the Swiss wheat collection.

To discover new potential candidate genes for mildew resistance, we focused on all regions detected having significant *k*-mer associations not overlapping with previously known *Pm* genes (Additional file 1: Fig. S12). Such comparison with known *Pm* genes combined with LD decay analysis allowed us to define new potential loci involved in powdery mildew resistance based on the CS genome. Defining borders of associated regions from a GWAS output can be difficult and is strongly influenced by linkage disequilibrium (LD) around the corresponding genomic region. For instance, a comparison between the *Pm2* and *Pm4* regions (Fig. 4A, B) shows that the LD landscape can vary significantly between different regions of the genome. Even though previous studies have calculated LD decay over entire subgenomes [41], we observe that large regions of high LD are present (Additional file 1: Fig. S11), making the half LD decay measure for our approach not suitable.

Several of the potentially novel loci we report are located on chromosomes where no *Pm* genes have been previously described. For all the rest, located on the same chromosome as a previously described *Pm* gene, we only considered those loci as novel that mapped much beyond the half-maximal LD thresholds described in Supplemental Fig. 11. As each region is a specific case and to allow users to explore the variation of LD for a region of interest, we developed a Shiny app (https://benjiapp.shinyapps.io/LD_plot/) (Additional file 1: Fig. S14) that displays the LD plot of a region of interest along with the *k-*mers of the region that are significantly associated with resistance. For the cases presented here, no big insertions/deletions were observed, and thus, for those regions, we extracted all protein sequences within 1 Mb around each detected peak. Nonetheless, we recommend following a case-by-case approach to define the extent of the region around a peak.

We then used BLASTp for all those genes to infer gene function. After several filtering steps (see “Methods” for details), Manhattan plots for each of the genomes combining the different isolates, as well as a summary table of the main associated regions, are presented in Additional file 1: Fig. S15. The table shows the presence of the main regions in the different genomes used as reference. As the genomic coordinates for each genome cannot be directly compared, the correspondence of the region of association between the genomes is arbitrary and some similar regions might not be the same in another genome. For in-depth analysis of candidates of choice, alignment between the genomes of the region of interest will be required.

As an example, we present a resistance-associated region located at the beginning of the short arm of chromosome 3D and was only detected when using Lancer, Renan, and CS as reference genomes (Fig. 4C). Its presence in these three genomes is not consistent with phenotypic observations: while Renan and Lancer are resistant to many of the tested powdery mildew isolates, CS is not resistant to any. To further explore this region, the 2 Mb around the most associated *k*-mer was extracted and aligned with all the genomes. This region is conserved between CS, Lancer, and Renan but not for the other genomes (Fig. 4D). Given that CS is not resistant, it is expected that haplotype diversity occurs in the region between Lancer/Renan and CS. Indeed, we observed such diversity within the first 500 kb of the region, which shows considerable variation between CS and Lancer, but is almost fully conserved between Lancer, Renan, and Norin 61 (Fig. 4D). While Lancer is resistant to 8 of 10 isolates, Renan is only resistant to CHE_96224, IRN_GOR5, THUN12, JPN_CHIKA, and TUR_1C, and Norin 61 is susceptible to all isolates. After extracting all the genes annotated in the region from the Lancer genome, we blasted them to all other genomes to find how conserved each of the genes were. The heatmap in Additional file 1: Fig. S17 summarizes the results of this analysis.

Each of the protein sequences has been also blasted to the NCBI database to extract possible functions. Thirteen genes are found to be NBS-LRR genes, although all of them are fully conserved between Lancer and Norin 61, including ±2 kb of the coding region. Because Norin 61 is fully susceptible such genes are likely not responsible for the resistance observed in Lancer. We found 10 genes present only in Lancer (Additional file 1: Fig. S17), out of those five are uncharacterized proteins, two are wax ester synthase like proteins, one is a fatty acyl-CoA reductase, one is a glucomannan 4-beta-mannosyltransferase, and one is a receptor like prot12. Waxes are part of the cuticle and protect different organs from biotic and well as abiotic stresses [42]. Even though such genes have not been described in resistance to powdery mildew, their presence/absence pattern between the genomes makes this gene family a solid candidate. Thus, using *k*-mer GWAS we detected new candidate genes and further analysis of the identified regions and the candidate genes will reveal a possible role of those genes in resistance.

*k*-mers allow to trace candidate genes across multiple genomes and examine their structural variation.

One intriguing region was identified when we mapped significantly associated *k*-mers with *Bgt* isolate CHE_96224 onto different wheat reference genomes using as basis the mapping to the SY Mattis genome to assign *k-*mers specific to *Pm2* and *Pm4* genes (Additional file 1: Fig. S18). Since SY Mattis carries *Pm2* and *Pm4*, we can tag the *k-*mers mapping at the region of *Pm4* and *Pm2* and predict their presence in other genomes. We would expect them to map onto other wheat reference genomes that also contain the *Pm2* and *Pm4* genes, as indicated by GWAS peaks in Landmark (*Pm2*) and Stanley (*Pm4*) (Additional file 1: Fig. S15). However, *k*-mers that are not associated with *Pm4* or *Pm2* (in black) form prominent peaks on chromosomes 3 A and 3D in Julius, Fielder, and Lancer and can reveal new potential candidate genes.

These *k*-mers were directly located within a Werner-like exonuclease gene (*TraesJUL3A03G01434660*). However, both Julius and Fielder are susceptible to CHE_96224. To explain this apparent contradiction, we investigated whether the Werner-like exonuclease gene exhibits copy number variation across wheat reference genomes. This gene is conserved in homeologous groups 3 and 7 in all wheat genotypes. However, its presence/absence pattern on chromosome 2B suggests a potential role in increased susceptibility to wheat powdery mildew (Fig. 4E). Specifically, the gene is present in the genomes of CS, Landmark, Norin 61, and Stanley—all susceptible cultivars—but absent in all resistant cultivars, Renan, Mace, and SY Mattis (Fig. 4F).

Interestingly, a Werner-like exonuclease coding gene has been identified in a DAP-seq (DNA affinity purification sequencing) experiment searching for TaZF binding sites. TaZF proteins have been implicated in powdery mildew resistance by recruiting both *Pm2a* and *AvrPm2* from the cytosol to the nucleus [25]. While future functional validation is required to confirm whether the Werner-like gene contributes to susceptibility, this case highlights how association mapping based on *k*-mers enables the examination of structural variation of candidate genes across different genomes.

### Using progenitor genomes allows the discovery of new associated regions with powdery mildew resistance

To find the location of the *k*-mers absent in all tested *Triticum aestivum* genomes, we mapped the reads to four progenitor genomes of wheat. The resulting Manhattan plots are represented in Additional file 1: Fig. S19. To compare with the location of the known *Pm* genes, we also blasted all the cloned *Pm* genes to the four genomes and their location is indicated in the Manhattan plot (Additional file 1: Fig. S19). In *Aegilops tauschii*, *Triticum urartu*, and *Triticum spelta*, we detected a region overlapping with the position of *Pm2* or its homolog on chromosome 5A. *Pm2* was originally described as a gene introgressed from *A. tauschii* into chromosome 2D [43]. For all four genomes, we detected a region at the end of chromosome 7 that tags the region of *Pm1* and *Pm60*, originally from *Triticum aestivum*, as well as *Triticum monocucum* and *Triticum urartu*, respectively*.* For all the other regions, we used the approach based on the blast described above to annotate the gene within the 1 Mb around each peak. After filtering the blast results, we ended up with 4, 6, 2, and 15, significantly associated regions containing candidate genes in *Aegilops tauschii*, *Triticum spelta*, *Triticum turgidum*, and* Triticum urartu*, respectively. Except for the significantly associated regions on chromosomes 7, 5, and 2, overlapping with *Pm1/Pm60* and *Pm2* and *Pm4*, respectively, all the other associated regions do not overlap with previously detected regions using the 10 *Triticum aestivum* genotypes as reference genomes. For example, new associated regions were detected at the end of chromosome 5D and 5 A in *Aegilops tauschii* and *Triticum urartu*, respectively, or at the start of chromosome 3D, 3 A, and 3A/3B for *Aegilops tauschii*, *Triticum urartu*, and *Triticum turgidum*, respectively (Additional file 1: Fig. S19). Therefore, the use of wild relative genomes as reference revealed additional potential resistance genes undetected in hexaploid wheats, suggesting that resistance genes were left behind or introgressed during breeding of hexaploid wheat.

*k*-mer GWAS allow the discovery of potentially new candidate genes for adult-stage resistance to wheat powdery mildew.

**Fig. 5:**
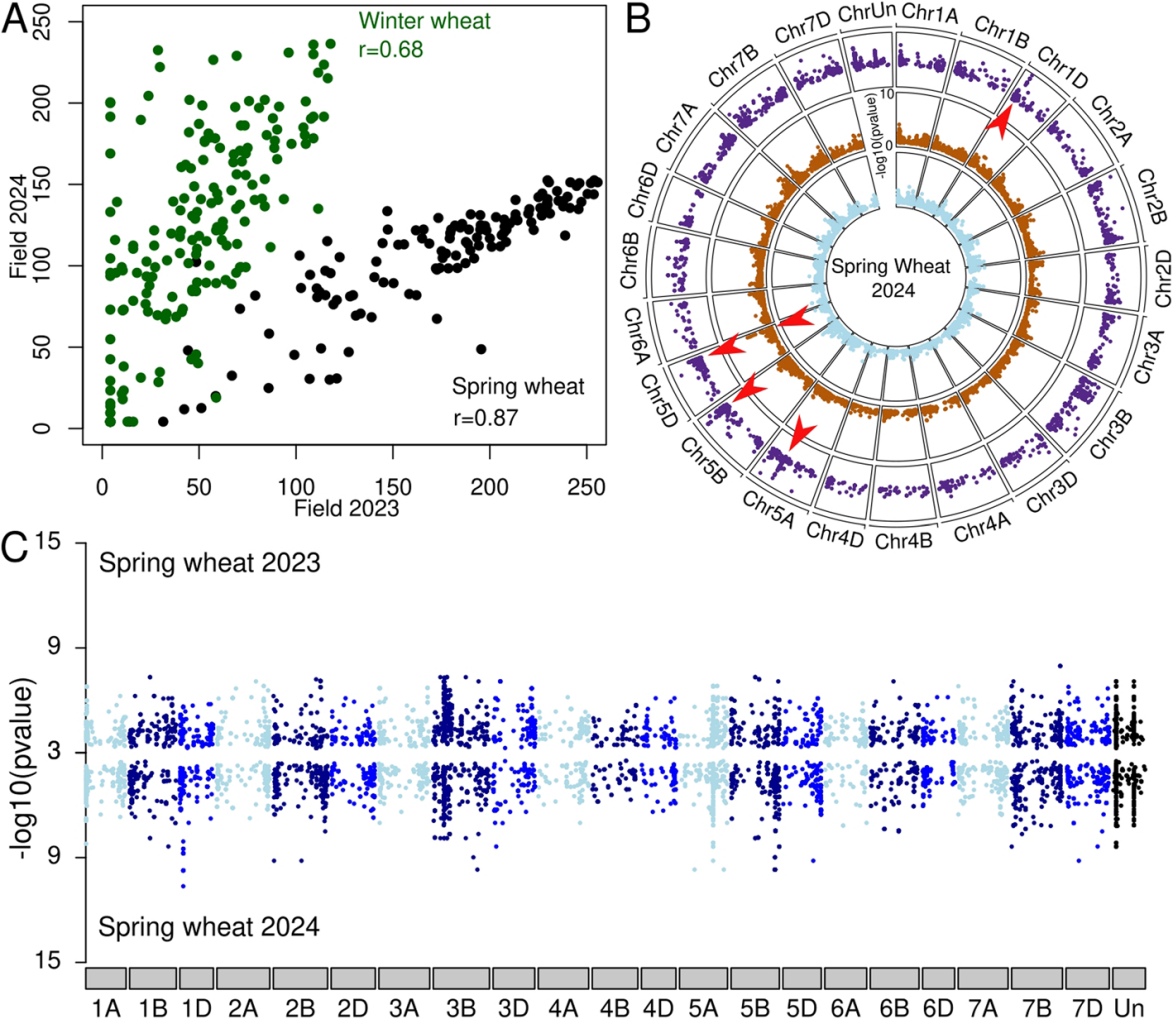
Genetic basis of wheat resistance at adult stage. **A** Correlation between the 2 years of field phenotyping for spring (black) and winter (green) wheat. *r* represents the Pearson coefficient. **B** GWAS using Spring Wheat 2024 for phenotypic values and for comparison of three genotype matrices. The inner circle is DArTseq SNP, the middle SNPs chip, and the outer *k*-mer GWAS. All SNPs are plotted for the two innermost circles, but only the *k*-mers above − log10(*p* value) of 3 are plotted in the outermost circle. Red arrows represent the main significantly associated regions. **C** Double Manhattan plot comparing *k*-mer GWAS from the 2 years of Spring wheat phenotyping. 2023 at the top and 2024 at the bottom. Only *k*-mer with a significance above − log10(*p* value) of 3 is displayed. The colors are representing the different subgenomes of wheat. Light, dark, and blue represent the A, B, and D genomes, respectively

We evaluated our panel under field conditions for adult plant resistance against powdery mildew over 2 years. As the panel included spring and winter wheat accessions and the AUDPC (area under the disease progress curve) values cannot be compared between these two groups, the collection was split in winter (276 accessions) and spring wheat (139 accessions), both showing high Pearson correlation coefficient (*r*) of AUDPC values, 0.68 and 0.87 between the two environments (years), respectively (Fig. 5A).

The identification of genomic regions associated with adult plant resistance in GWAS studies is challenging. Such resistances often involve several genes, reducing their respective association with the observed phenotypes and making it more difficult to be detected by GWAS. To test whether our *k*-mer approach outperforms standard pipelines, we performed a GWAS for adult-stage resistance comparing the *k*-mer and the two SNP matrices using field phenotypes. Here, we focus on the Spring Wheat 2024 trial as an example (Fig. 5B). We identified a significantly associated region at the end of chromosome 5D (around 500 Mb) using the SNP set obtained through DArTseq. The associated region on chromosome 5D does not overlap with the region found using the other matrices (SNPs chip and *k*-mers). Moreover, the *k*-mer GWAS identified multiple, narrower associated regions on chromosomes 5 A, B, D, and 1D. To compare the GWAS results between two different years, we aligned two Manhattan plots together (Fig. 5C). We found that most regions consistently appeared in both years even though some regions only passed the significance threshold in one of the 2 years. We observed a similar pattern for the winter wheat data where the same associated regions were detected in both years (Additional file 1: Fig. S20). This is expected considering the high correlation between the phenotypes for the 2 years.

Adding the 10 genomes of *Triticum aestivum* as a reference, we again detected additional associated regions present in only one or few genomes. For example, one region on chromosome 4B was only present when Julius’ genome was used as a reference (Additional file 1: Fig. S21). We conclude that, despite a limited number of accessions and challenging field phenotyping for adult plant resistance to powdery mildew, using *k*-mers and multiple reference genomes greatly improved the precision and power of GWAS in detecting resistance loci compared to standard pipelines.

## Discussion

To fully leverage germplasm collections for valuable alleles that can enhance crop resilience, comprehensive genetic and phenotypic characterization is crucial—an endeavor that has only recently begun to be undertaken [9, 30, 44]. However, recovering rare alleles from the vast genetic landscape of global diversity panels can be challenging [45, 46]. Alternatively, the use of local or regional diversity panels, better adapted to local agroecosystems [47, 48], may be more suitable for specific breeding applications. In our work here, we address this gap by assembling a collection of 461 wheat landraces and old cultivars, representative of the wheat gene pool in Switzerland, to characterize landscape of resistance to wheat powdery mildew, a persistent disease in Swiss wheat fields and that has evolved across decades [49].

While some association mapping studies in search of all-stage powdery mildew resistance are limited by the use of a single mildew isolate [50–52] or just a few with similar virulence patterns [53], our study significantly broadens the analysis by testing the disease resistance to ten wheat powdery mildews from diverse geographic regions, each exhibiting contrasting virulence patterns based on the disease responses of 37 *Pm*-tester lines. On average, 80 accessions (17% of the collection) were resistant to single *Bgt*. This contrasts to similar studies evaluating seedling resistance to wheat powdery mildew. For example, Li and colleagues [53] tested 1292 accessions, where only 4% were resistant to the tested *Bgt* isolate, or [51], who reported overall susceptibility at the seedling stage among 8316 winter wheats of the German Federal ex situ gene bank. Our results underscore the suitability of our collection as a source of resistance to wheat powdery mildew.

By comparing response patterns derived from the interaction between wheat landraces and the set of 10 *Bgt* isolates with known virulence/avirulence spectra based on 37 *Pm*-tester lines, the presence of *Pm1*, *Pm2*, *Pm4*, and *Pm60* resistance genes could be postulated. Further, the presence of *Pm2* and *Pm4* could subsequently be confirmed using diagnostic molecular markers. Of note, the 28 landraces fully resistant to the 10 tested *Bgt* isolates have the same pattern as the *Pm13* and *Pm36* tester lines. Both *Pm13* and *Pm36* genes have been recently cloned and encode kinase-fused resistance proteins [54, 55], a new resistance gene family private to Triticeae that provides resistance to different fungal diseases in major cereal crops [56]. Further work is needed to confirm if *Pm13* and *Pm36* resistance genes are responsible for the “all isolate” resistance observed in the 28 landraces, or if novel, unknown *Pm* genes are causing this broad-spectrum resistance. If the presence of these resistance genes is confirmed in hexaploid wheat landraces, they could be used more broadly in breeding programs. *Pm13* was cloned from *Ae. longissima* [54], while *Pm36* presence has only been documented in a few wild emmer wheat accessions [55], making its practical use in breeding programs challenging. As landraces are sexually fully crossable with bread wheat, these resistance loci could be directly cross-bred into modern cultivars, sidestepping long and laborious backcrossing with wild relatives like *Ae. longissima*.

As the bimodal-like distribution of seedling response to wheat powdery mildew suggested the presence of major genes controlling resistance, we performed a GWAS with genotyping data generated with the Illumina Infinium 15K wheat SNP array (TraitGenetics GmbH, Gatersleben, Germany). Association mapping analysis revealed 53 significant SNPs, pinpointing seven genomic regions associated with all-stage resistance, two of them corresponding to the *Pm1* (or *Pm60*) and *Pm2* resistance genes. This apparent limited capacity of detecting resistance loci may be explained by the intrinsic nature of the SNP-chip. First, the chip can only detect SNPs. However, SVs such as insertions, deletions, duplications, CNVs, and translocations have been reported to underlie disease resistance phenotypes [57]. Second, SNP-chip design relied on a single reference genome, Chinese Spring, and a few accessions, limiting the SNP set that can be detected [58].

To overcome these limitations, we further genotyped the collection with DArTseq technology, a genotyping technology that is not biased by reference genomes, as it sequences restriction fragments produced by restriction enzyme-mediated genome complexity reduction, which theoretically increases its capacity to detect loci of interest compared to the SNP-chip [14]. Using such data and CS as a reference genome, a SNP matrix containing 10,068 SNPs was generated. The use of these two SNP matrices resulted in significant association with wheat mildew resistance overlapping with the presence of known *Pm* genes like *Pm2* and *Pm4* (Fig. 3) [27, 28]. Interestingly, although the SNP matrix derived from DArTseq contained less SNPs compared with the SNPs chip, it resulted in sharper peaks. Such associated regions contain SNPs with a high proportion of missing data and only when including SNPs with 80% missing data, all SNPs were included for GWAS and more association could be detected. This is not surprising knowing that wheat genomes contain many introgressions [40] that would result in such a pattern.

In order to avoid the bias of the reference genome, we generated a matrix of *k*-mers from the raw data of DArTseq. This led to 176 million unique *k*-mers of length 31 bp. Such *k*-mers can tag SNPs, as well as other structural variations [21], and it is known that, especially in plant genomes, an important part of genetic diversity is due to structural variations in different forms, such as presence/absence variants (PAVs), CNVs, insertions, or deletions [59]. The disadvantage of using *k*-mer is that the location in the genome is not known. When using Illumina sequencing data, reads containing *k*-mers can be assembled into larger segments and subsequently Blast or sequence alignment can be used to find the corresponding gene [21]. However, the nature of DArTseq data does not allow such an approach. To circumvent this limitation and being capable of comparing with the SNP GWAS, we mapped the DArTseq raw reads to CS. We could detect all the associated regions already detected using SNP matrices as well as new ones on chromosomes 2 A and 5D. However, only 68% of the significantly associated *k*-mers could be mapped using the reference genome Chinese Spring. The discrepancy with the number of reads mapping (97.3%) again points towards the fact that introgressions are well-known to harbor genuinely new alleles of agronomic interest [38]. To overcome this, we used multiple reference genomes: ten bread wheat genomes and four genomes from the progenitors of wheat to capture as much variation as possible. Still, the main disadvantage of *k*-mer-based GWAS approaches is the lack of standardized methods for conducting such analyses. This is in sharp contrast to user-friendly programs like TASSEL [60] or GAPIT [61] that have been developed for SNP-based GWAS pipelines. To apply the *k*-mer-based association mapping analysis to multiple genomes, we developed a pipeline. With this, we increased from 11,570 significant *k*-mers when using CS as a reference genome to 15,712 significant *k*-mers. This translated into the identification of 27 potential additional new regions associated with resistance to powdery mildew. The inclusion of more reference genomes allowed us to map 25% more *k*-mers reaching 93% of all the significantly associated *k*-mers detected using GWAS (Fig. 3). Among the 27 regions, we focused on two of the most promising candidates: a gene annotated as a Werner-like exonuclease and a wax ester synthase gene in chromosomes 2B and 3D, respectively. Notably, none of the two regions was revealed when using the SNP matrices and also does not overlap known Pm loci, which highlights the higher discrimination power of the *k*-mer-based approach. This also suggests that these genes’ presence or absence may be attributed to introgressions.

In humans, Werner-like exonucleases have been shown to degrade DNA in a structure-dependent manner [62]. In *Arabidopsis thaliana*, Werner-like exonucleases have been shown to interact with the Ku heterodimer which is required for the non-homologous end joining pathway of DNA repair [63]. However, no studies to date report their involvement in plant immunity. The correlation with the phenotype shows that the presence of the gene is associated with susceptibility to four *B. graminis* isolates, CHE_96224, IRN_GOR5, JPN_CHIKA, and TUR_1C as well as THUN12 (Fig. 5). The genomes of CS, Landmark, Norin 61, and Stanley contain the two copies of the Werner-like exonuclease gene on chromosome 2B, while the other genomes do not have any. Susceptibility factors are rare, but some prominent examples have been described in cereal immunity. For instance, the well-known natural mutation of the *Mlo* gene in barley provides broad-spectrum resistance against barley powdery mildew [64], while the *pi21* recessive mutation is linked to long-lasting resistance to rice blast [65]. More recently, genome-edition of the wheat *Mlo* orthologue has proven to confer resistance to wheat powdery mildew without growth penalties [66], while the inactivation of a target for rust effectors, the wheat kinase *TaPsIPK1*, confers broad-spectrum resistance to rust fungi. *CRISPR*/Cas9 mutations of the two copies of the gene on chromosome 2B would clarify if the Werner-like gene is a susceptibility factor, which if proven, would be the second report of a disease susceptibility gene to wheat powdery mildew, opening up new avenues to control the disease.

The region around 5 Mb of chromosome 3D is present in four out of all the genomes tested, namely CS, Renan, Norin 61, and Lancer. Such patterns correspond to a possible introgression that is strongly associated with resistance to powdery mildew. As the resistance pattern observed on Renan genome can be fully explained by the presence of a functional allele of *Pm4*, and CS as well as Norin 61 is fully susceptible, we focused on genes solely present in Lancer. We found that 3 of the 12 genes present in Lancer in these regions are involved in wax synthesis. Even so they are not known to be directly involved in resistance to powdery mildew, waxes of the cuticle are forming a physical and chemical barrier to biotic and abiotic stresses [42]. Further functional and molecular analyses are required to confirm the possible role of such genes in plant defense.

Adult plant resistance to diseases is genetically complex, usually controlled by several genes associated with genomic regions called quantitative trait loci (QTL), resulting from the effect of each QTL acting—with small or larger effects—leading to a partial phenotypic resistance. Detection of minor-effect QTLs is highly challenging [7]. To further test the detection power of our *k*-mer-based GWAS approach over the SNP-based approach, we performed association analysis using adult plant resistance to wheat powdery mildew collected under field conditions over two seasons. We found that when using SNP matrices, no significantly associated regions to mildew resistance were detected, while the use of our *k*-mer GWAS allowed the detection of multiple regions of interest. With our approach, we show that the capacity to detect those small QTLs is achieved compared to the commonly used SNP-based approaches. Implementing our pipeline to future field studies would result in more accurate and larger detection of QTLs, allowing breeders to accumulate major- and minor-effect loci that can be used for the improvement of wheat powdery mildew (and virtually any other disease) resistance.

The *k*-mer-based pipeline presented here offers several advantages over conventional SNP-based association mapping analysis. First, *k*-mers allow for the assessment of almost all types of variations [21]. While SNP matrices can only detect SNPs, *k*-mers can interrogate diversity given by SVs, such as large indels or introgressions. This led to the discovery of loci associated with resistance that would have been missed using SNP matrices. Second, by utilizing *k*-mers directly from raw sequencing data, we could bypass error-prone stages of variant discovery and genotyping, thereby facilitating the identification of causal variants. Third, the inclusion of multiple reference genomes notably expanded the number of potential loci associated with resistance by interrogating broader diversity [20]. All these improvements translate into greater efficiency on the same phenotypic and genotypic basis, both expensive and labor-intensive to get.

With the increase in the number of genomes being sequenced, we foresee that our approach can become very popular as pan-genome GWAS using SNPs has not yet been implemented. Our *k*-mer GWAS approach proves to be a better choice than relying on SNP markers as *k-*mers can more accurately represent genome diversity, and detailed comparison between available reference genomes would lead to the identification of useful alleles and allele stacking strategies to sustain resistance breeding activities.

## Conclusions

We present a scalable *k*-mer-based GWAS framework that leverages multiple reference genomes to capture variation and improve trait mapping in complex plant genomes such as hexaploid wheat. By accessing a broader spectrum of genetic diversity—including structural variants and introgressions—our method surpasses traditional SNP-based approaches in both resolution and discovery potential. This enhanced mapping capability emphasizes the value of landraces and historical cultivars as rich reservoirs of genetic variation. As the number of high-quality reference genomes continues to expand, our approach will enable a more comprehensive exploration of pan-genomic diversity and support the identification of alleles relevant to a wide range of agronomic traits. These advances provide a flexible and efficient foundation for trait dissection and the informed use of germplasm in modern crop improvement programs.

## Methods

### DNA extraction and genotyping

The 461 accessions from the Swiss collection have been genotyped using Diversity Arrays Technology Pty Ltd (http://www.diversityarrays.com/) for sequencing and marker identification as a batch of the AGENT project. All the data have been uploaded and can be found as BioProject PRJEB81686. Seeds were provided by the Swiss gene bank at Agroscope. Individual plants for DNA extraction were grown in a climate chamber cycled at 20 °C/16 °C, 16/8 h photoperiod with 60% relative humidity. Two segments of the first leaf (each approximately 3 cm long and 0.3 to 0.5 cm wide) were placed in 2.2-ml tubes with two 4-mm glass beads (ROTH). Samples were frozen in liquid nitrogen and ground with a Geno/Grinder (SPEX SamplePrep) at 1500 rpm for 1 min. Subsequent steps for DNA binding, washing, and elution were carried out using the automated KingFisher™ Apex Purification System (ThermoFisher) as described in [67, 68]. Purified DNA was dissolved in 10 mM Tris HCl pH 8.0 and shipped frozen with a blue icepack.

### SNP-chip generation

Plants were genotyped using an Illumina Infinium 15K wheat SNP array (TraitGenetics GmbH, Gatersleben, Germany) composed of 13,006 SNPs. The sequences and the position of the molecular markers on the IWGSC CS RefSeq v2.1 were retrieved from the 90K iSelect (Kansas State University, Manhattan, USA) and the Breeders’ 35K Axiom® arrays (Axiom, Santa Clara, USA) from which originated the Illumina Infinium 15 wheat SNP array. This led to unambiguous positioning of 11,983 markers out of the 13,006 SNPs. One thousand twenty-three markers remained with unclarified physical position, either because (i) originally not mapped to any chromosome (41 markers) or (ii) mapped to two or more positions often on the three wheat homoeologous chromosomes (982 markers). These unmapped or not precisely mapped markers were blasted against the IWGSC Cs RefSeq v2.1 using GrainGenes online tools (available at https://wheat.pw.usda.gov/GG3/, accessed 12/08/2024). The physical location associated with the lowest *E* value was retained. This leads to unambiguous positioning of 981 additional SNP on the reference genome sequence representing a total of 12,964 SNPs. Moreover, 415 SNP markers containing more than 25% of missing information were trimmed-off the SNP matrix. The remaining SNP table was imputed using Beagle 5.4 [69] and 733 SNPs with a minor allele frequency (MAF) of less than 5% were removed. After the different cleaning operations, 882 SNPs (on a total of 11,816 SNPs) could be reassigned by BLASTING to unambiguous position IWGSC CS RefSeq v2.1 and could serve as a genotypic table obtained after 15K Chip genotyping.

### Mapping

The fastq files containing the sequencing data from DArTseq were mapped to the different genomes using BWA mem [70]. While duplicated reads were marked by Markduplicates from Picard (v1.101; http://broadinstitute.github.io/picard/), samtools (v1.13) was used for file format conversions like sorting and indexing [71]. The transformation from the obtained “.bam” files to “.bed” files was done using BEDtools v2.30.0 [72]. Such files were further processed for different analyses using R. The scripts are available on GitHub.

### Read processing and variant calling from the DArTseq data

Adapter sequences from raw reads obtained from DArTseq sequencing were trimmed using cutadapt (v1.9.1) [73] with a minimum read length of 30 bp. Reads were aligned against the hexaploid wheat reference genome assembly cv. Chinese Spring (RefSeq v2.1) [33] using BWA-MEM (v0.7.15) [74] with default parameters and the output was converted to binary alignment map (BAM) format using SAMtools (v1.3) [71]. BAM sorting was performed using NovoSort (V3.06.05). Variant calling was performed using the mpileup and call functions with the multiallelic-caller (-m) from BCFtools (v1.12) [75] with a minimum read quality (-q) cutoff of 20 and retaining allelic depth and the number of high-quality bases (-a AD,DP) for variant sites. SNPs were further filtered for minimum QUAL ≥ 40, minimum read depth for homozygous calls ≥ 2, minimum read depth for heterozygous calls ≥ 4, and a minimum presence rate of 80% using a custom awk script.

### Population structure analysis

As input, we used a SNP file (vcf) generated from the DArTseq data, containing 10,068 SNPs. We used the ADMIXTURE program [76] to analyze the data. To generate the different plots presented in Additional file 1: Fig. S7, we used R following the pipeline available at https://github.com/speciationgenomics/scripts/blob/master/plotADMIXTURE.r.

The Swiss collection and *Pm*-tester lines.

The Swiss collection consists of 461 accessions: 139 are classified as spring wheat and 276 as winter wheat; the rest are of unknown growth habit. Accessions were named after villages in Switzerland. Their location of each accession has been extracted based on the corresponding village name in Switzerland. These are approximations, as no precise locations are available for those accessions. Full details of the accessions are available in Additional file 2: Table S2. The set of tester lines consists of 37 different lines that carry 24 different *Pm* resistance genes. The genotyping of the *Pm4b* and *Pm2* across all the accessions was performed by PCR using haplotype specific markers as described by [27] and [31], respectively.

### Powdery mildew isolates and phenotyping infections

Prioritizing genetic diversity (from section “Powdery mildew isolates genetic analysis”), and maximizing contrasting virulence/avirulence patterns on Pm-tester lines, we selected the following available and alive powdery mildew isolates: CHE_96224, KAZ_1b, JPN_CHIKA, GRB_JIW2, ARG_4_2, TUR_1C, and POL_3 are described in [23]. Additionally, CHE_97251 [27], THUN12 [77], and IRN_GOR5 [78].

The infection of the leaf segments was done as described in [30]. Disease assessment was conducted 8–10 days after inoculation using a discrete percentage scale, based on visual evaluation by human eye, where 0% no visible disease symptoms and 100% represented complete coverage of the leaf with sporulating colonies [79]. Accessions were considered resistant with a score below 20 and susceptible with a score above 20 (Leber et al.). Some examples of samples are shown in Fig. 1A.

### Powdery mildew isolate genetic analysis

The raw Illumina reads were filtered, mapped, and haplotype-called to the CHE_96224 reference genome as previously described [23]. Following the same methods from before, the generated SNPs were filtered with vcftools (v0.1.16) with the following parameters: –minDP 3, –maxDP 1000, –maf 0.01 (minor allele frequency), –max-missing 0.999, excluding all SNPs on chromosome Unknown, removing InDels, and keeping only biallelic SNPs. In order to look at the genomic diversity of the wheat powdery mildew isolates, we performed a PCA on all the available isolates and then excluded the populations for which we did not eventually choose any representatives (i.e., CHN, AUS, and USA wheat mildew populations), resulting in 265 isolates. We performed the PCA analysis of these isolates using vcftools (v0.1.16). We then visualized the PCA via the R packages tidyverse (v2.0.0), ggplot2 (v3.5.1), ggrepel (v0.9.5), and rcartocolor (v2.1.1) [80–83]. The raw sequences of the datasets used in this study can be found in the SRA (Short Read Archive) of the National Center for Biotechnology Information (NCBI) under the accession project numbers found in the papers: [23, 84] BioProject PRJNA625429.

From the original dataset containing 389 wheat powdery mildew isolates, we kept only the nine isolates of interest using vcftools (v0.1.16). We then used plink2 (v2.00a3 64-bit (17 Feb 2020)) with the parameters “–indep-pairwise 50 10 0.1” to find the positions of SNPs that were not very close to each other. Next, we kept only the pruned SNPs using the “—extract” parameter in plink1.9 (v1.90b6.16 64-bit (17 Feb 2020)). Finally, we used plink1.9 with the parameter “—genome” to create an identity-by-descent report (IBD) between these isolates. The analysis was conducted using nine isolates (excluding THUN_12 as it is a hybrid) and ended up with 93,652 SNPs (excluding InDels, Chr-Un) and keeping only biallelic SNPs, and including only sites with minor allele frequency ≥ 0.01 and a proportion of missing data > 0.999. LD pruning as described above was also applied.

### Field phenotyping

Field setup and phenotyping were performed as described in [85]. All accessions were sown for Winter wheat 2023: 11.10.22, Spring wheat 2023: 2.3.23, Winter wheat 2024: 11.10.23, and Spring wheat 2024: 26.3.24. In short, each accession was planted in 4 replicates/blocks of 1.5 × 1 m. Those blocks were randomized across the field. Powdery mildew scoring was performed as in [86, 87] over the spring and summer years 2023 and 2024. In 2023, spring and winter wheat were phenotyped at 6 and 4 time points over 36 and 20 days, respectively. In 2024, 5 and 6 time points spanning 21 and 42 days for spring and winter wheat, respectively (Additional file 1: Fig. S22).

### Genomes used as references

For each of the 10 *Triticum aestivum* as well as the 4 progenitor genomes used as reference in this study, the full genome sequence as well as the annotation as gff3, and the protein sequence of the genes were downloaded from https://plants.ensembl.org/info/data/ftp/index.html. The genomes used are as follows: from *Triticum aestivum*: Julius, Renan, SY Mattis, Lancer, Chinese Spring, Fielder, Jagger, Landmark, Mace, Stanley, Norin 61 and *Triticum urartu*, *Triticum turgidum*, *Aegilops tauschii*, *Triticum spelta.* A list of all the genomes with the links to the genome sequence/annotation and protein sequence can be found in Additional file 2: Table S6.

Generating *k*-mers.

DArTseq produces a high coverage of reads, but for short regions, the first step before generating the *k*-mers was to remove all the perfectly duplicated reads. The program Clumpify (part of the package bbmap) v39.00 [88] was used, with default parameters (zl=9 and dedupe=t). This decreased the number of retained reads by about half. This was done as two identical reads will produce the same *k*-mers.

Based on the pipeline developed by [21], *k*-mers of length 31 bp were generated from the filtered fastq files for each accession using KMC v3 [89]. All parameters were kept to default except for the -Ci, which sets the threshold for how many times a *k*-mer needs to occur to be counted. Values of 1, 2, and 3 were tested (Additional file 2: Table S3) and similar proportions and associated regions were found for all. We used the 5% family-wide threshold to select the significant *k*-mers. The adapted pipeline can be found on https://github.com/benjj212/Kmer_GWAS_AGENT.

### GWAS

*K*-mer GWAS was performed on 10 traits corresponding to the 10 *Bgt* isolates following the methodology described in [21]. This pipeline uses GEMMA with a linear mixed model, correcting for population structure using a kinship matrix. The default minor allele count (MAC) of 5 was used. For the SNP GWAS, the GEMMA software was used [90] with option -lmm 2 and -maf 0.05. The Manhattan plots and QQ plots were generated using the package qqman (version 0.1.9) [91].

*k*-mer analysis.

The *k*-mers passing the 5% threshold were used for the 10 isolates, while all *k*-mers were used for the field dataset. These include all *k*-mers that exceed the − log10 threshold for the 5% family-wise error rate [21]. The number of *k*-mers as well as the − log10 threshold for each isolate is presented in Additional file 2: Table S3. From those *k*-mers, the list of reads names containing the corresponding sequences was extracted from the raw fastq files using the fetch_reads_with_kmers-master from https://github.com/voichek/kmersGWAS. The adapted pipeline is available at https://github.com/benjj212/Kmer_GWAS_AGENT. Using the read names, the bam files were filtered to retain only those specific reads. The filtered bam files were then transformed to BED format using the bamtobed function from the bedtools package [72]. Files containing the sequence of each significant *k*-mer, their *p* values, and the coordinates of the corresponding genomes were used for the generation of the Manhattan plot, as well as downstream analyses.

### SyRI

First, the different Fasta sequences were aligned using Minimap2.1 [92] with -ax asm5 -eqx to generate a.sam file. Once the alignments were generated, we used SyRI 1.6.3 [93] to generate the comparison files and detect the different types of structural variants between the two sequences of interest. We then used the function plotsr 1.1.1 [94] to generate the output plot. An example of such a pipeline can be found at https://github.com/benjj212/Kmer_GWAS_AGENT.

### Map/plots correlations/UpSet plot

All maps and plots were generated using R. Scripts used to generate the plots from the different figures are available on https://github.com/benjj212/Kmer_GWAS_AGENT. For some of the Manhattan plot, the package CMplot have been used [95].

### Shiny app for data visualization

Manhattan plot: https://benjiapp.shinyapps.io/Manhattan_plot/. Plot for the phenotype distribution: https://benjiapp.shinyapps.io/Map_agent_pheno/. Package of the Shiny app with all the data are also available on https://github.com/benjj212/Kmer_GWAS_AGENT. The LD_plot, https://benjiapp.shinyapps.io/LD_plot/, is based on the LDheatmap [96] package and the snpStats [97]. Screenshot with explanation is presented in Additional file 1: Fig. S14.

### Candidate gene selection

For each genome, we combined coordinates of reads containing *k*-mers with the *p* value from each isolate to detect the main peaks and generate Manhattan plots. Going through the genomes with a window of 1 Mb, all the windows containing at least 10 significant *k*-mers were extracted. Then from each region, all the protein sequences of the annotated genes within the region were saved. Each of the protein sequences was blasted to the plant NCBI database (taxid:3193). The blast has been automated using a Python script available on https://github.com/benjj212/Kmer_GWAS_AGENT. For each blast, the first two results were extracted and listed. The regions known to contain already characterized Pm genes were then tagged. The different steps are graphically explained in Additional file 1: Fig. S23.

## Supplementary Information

Additional file 1: Supplemental file, Figures S1–S22.

Additional file 2: Supplemental file, Tables S1–S8 information.

## Data Availability

The DArTseq raw sequencing data used in this study have been deposited on NCBI under the BioProject ID PRJEB81686 [98]. All scripts are available at https://github.com/benjj212/Kmer_GWAS_AGENT and are cited in reference [99]. The published sequencing data used for the powdery mildew isolates population analysis are available under the BioProject PRJNA625429 [23]. The source code of the three Shiny apps developed for this publication is available in references [100–102]. No additional scripts or software were used beyond those mentioned in the Methods section.
