# Supplementary material for: k-mer-based GWAS in a wheat collection reveals novel and diverse sources of powdery mildew resistance
Source: Genome Biol. 2025 Jun 18;26:172. doi: 10.1186/s13059-025-03645-z (PMC12175386; doi:10.1186/s13059-025-03645-z)
Supplement: Supplementary file 1 — Additional file 1: Supplemental file, Figures S1–S22. [file 13059_2025_3645_MOESM1_ESM.docx]

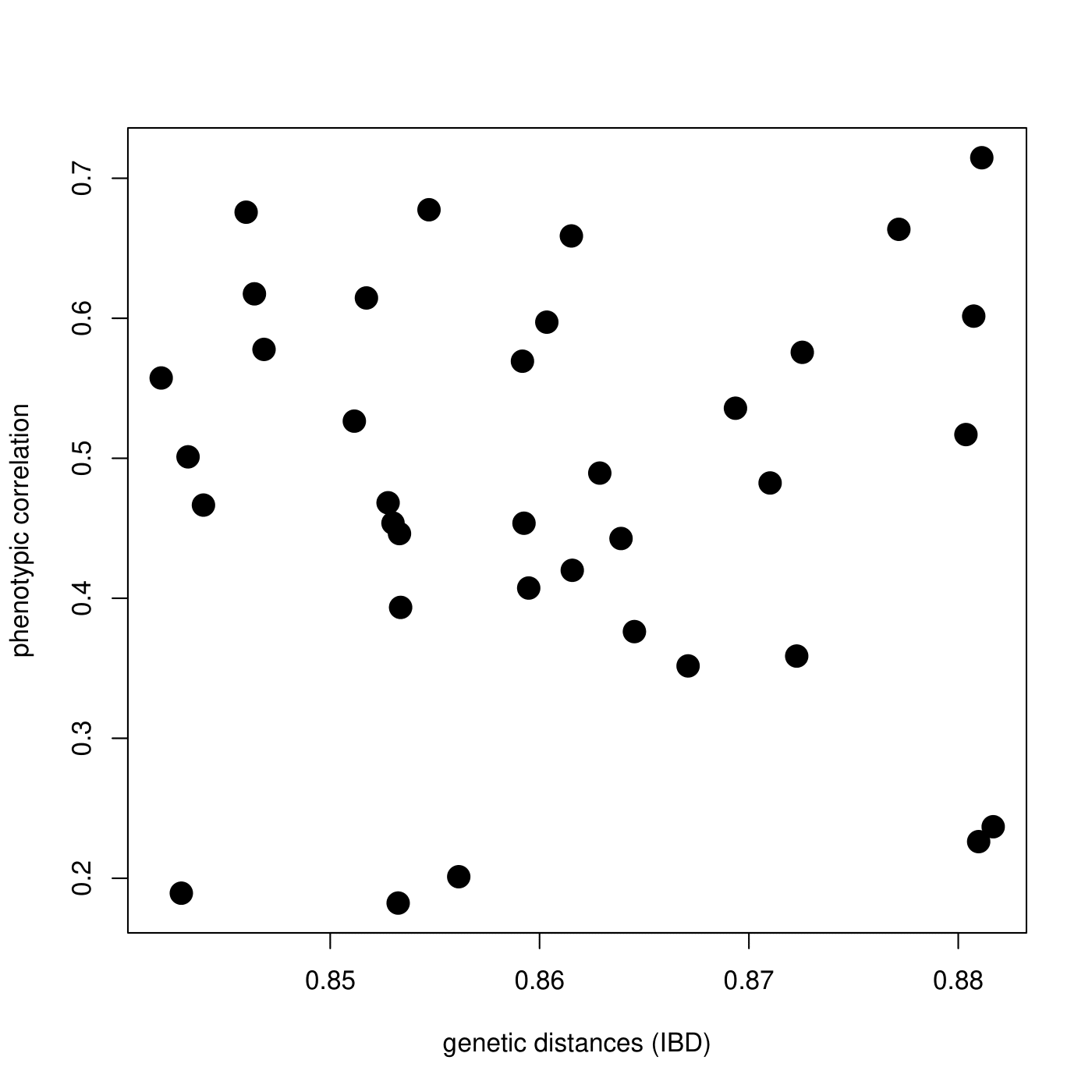


**Fig S1:** Comparison between genetic distance and phenotypic correlation among the *Bgt* isolates. Every dot represents a unique combination of two *Bgt* isolates. The x-axis represents their identity-by-descent (IBD) distance, while the y-axis represents the phenotypic correlation between two isolates based on the response of 461 accessions (as shown in Figure 2D).


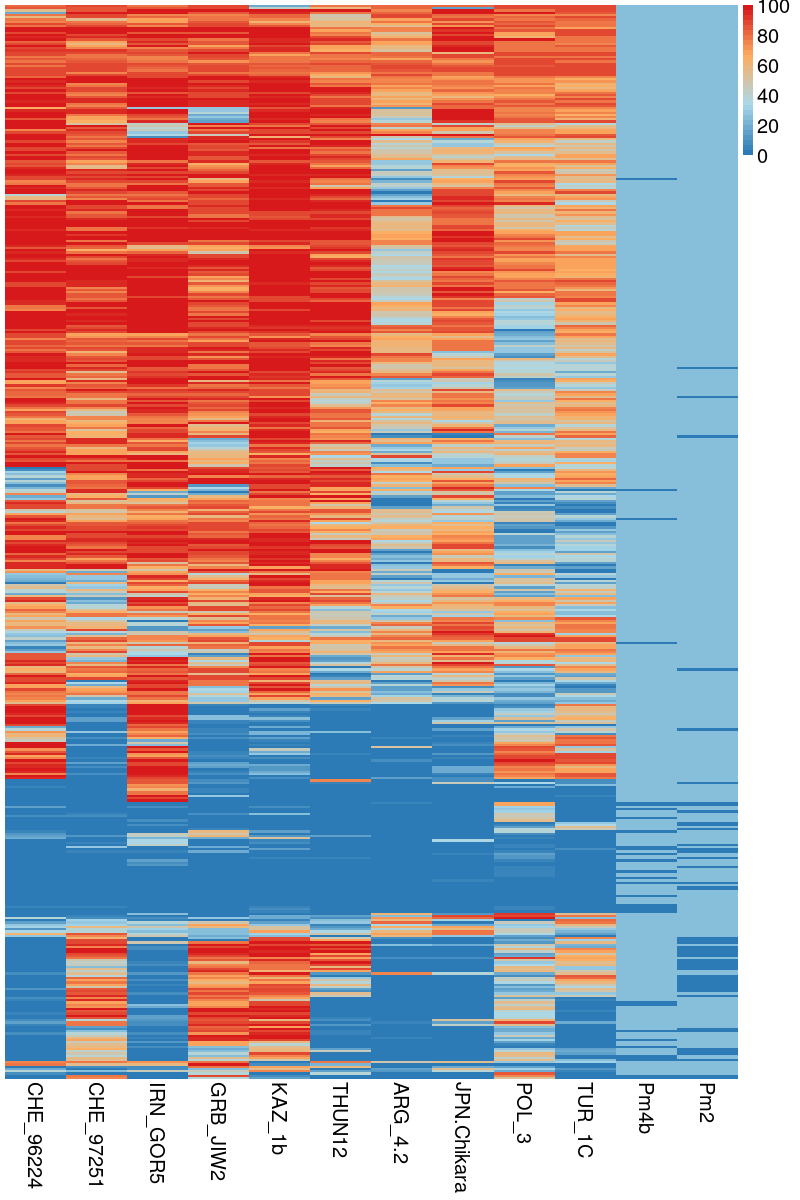


**Fig S2:** Heatmap showing the resistance pattern across the 10 *Bgt* isolates for all the accessions of the Swiss collection together with the corresponding genotype of *Pm4b* and *Pm2* for each accession. For the genotyping columns (*Pm4b* and *Pm2)*, green represents the presence of the gene.


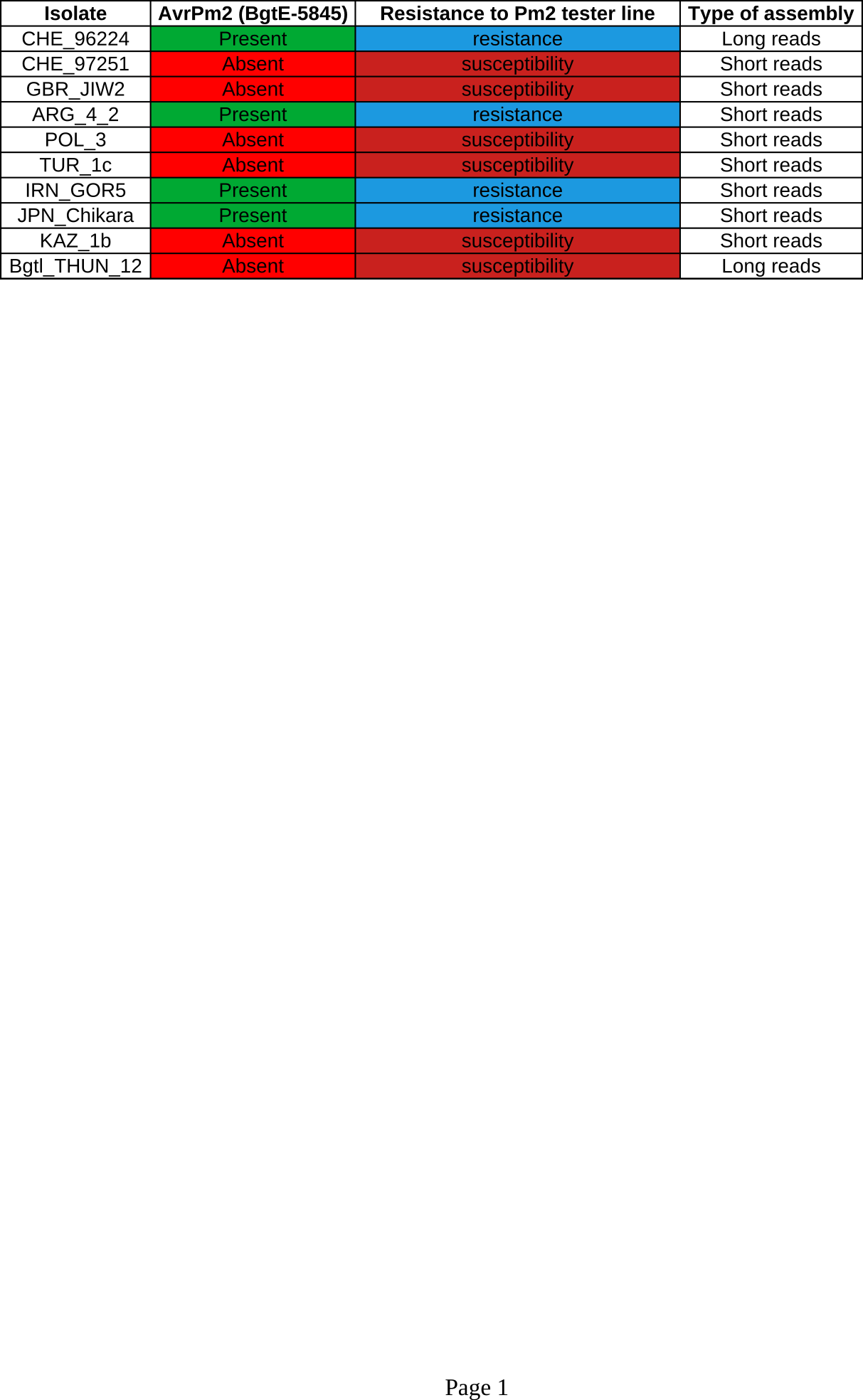


**Fig S3:** Presence/absence of the mildew effector *AvrPm2* in the 10 *Bgt* isolates used in this study. The *BgtE-5845* gene (*AvrPm2*) was used as a query for blast analysis. The third column represents the resistance or susceptibility pattern of the *Pm2* tester line to the corresponding *Bgt* isolate as presented in Figure 2B. The last column indicates the type of genome assembly used.


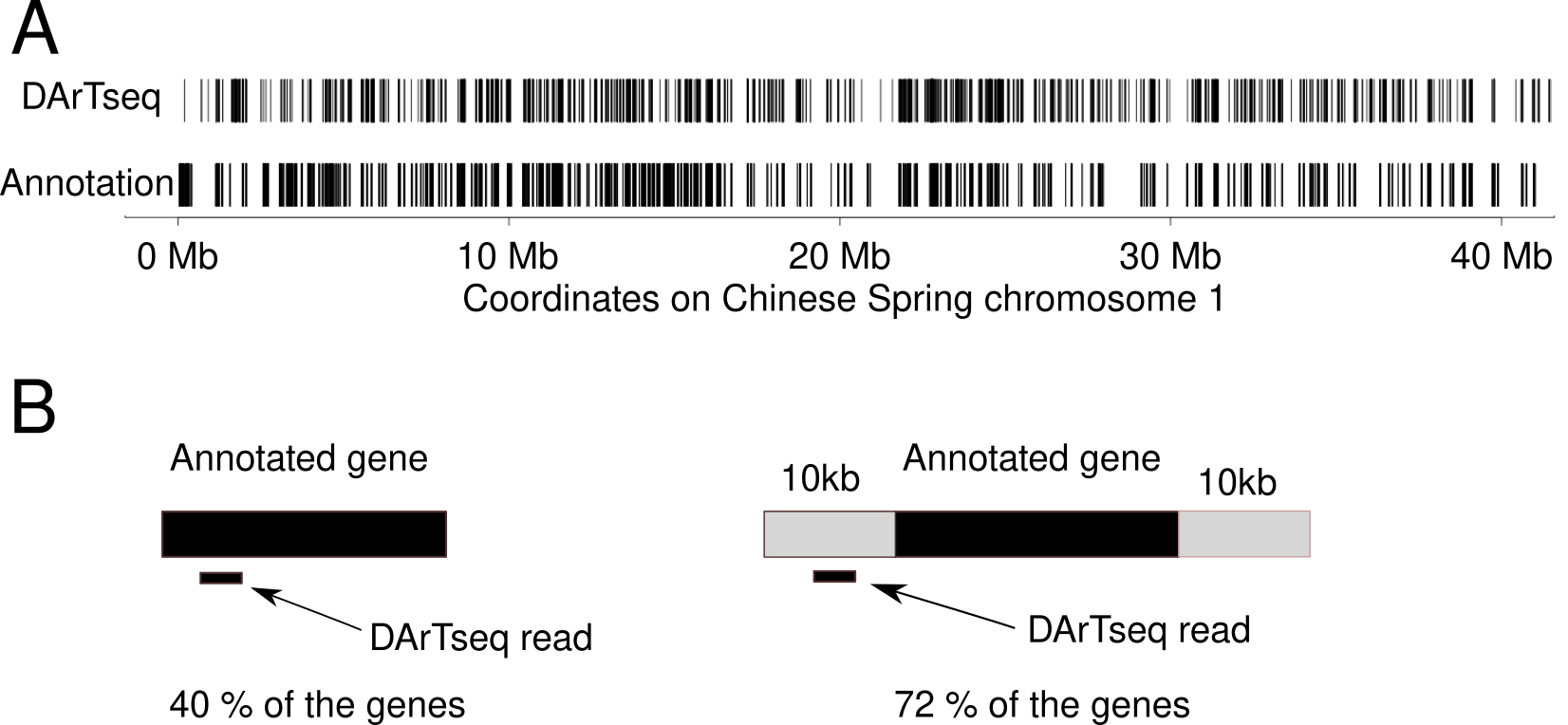


**Fig S4:** Evaluation of the mapping distribution of the DArTseq data. A: DArTseq reads mapping to Chinese Spring chromosome 1 compared with the gene annotation of the same genome. B: Proportion of DArTseq mapping within an annotated gene or within +/- 10kb of an annotated gene.


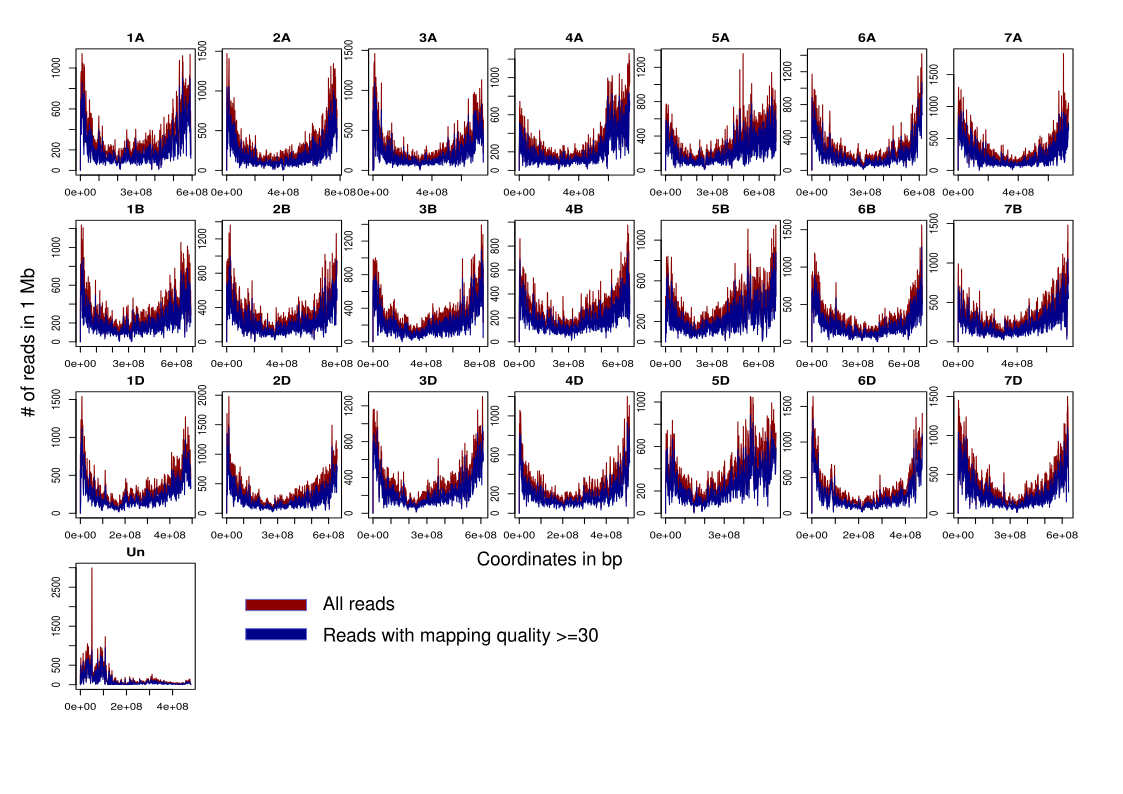


**Fig S5**: Profiles of reads mapping density across the 21 chromosomes of the Chinese Spring reference genome. Un: represents the unassembled contigs. All the reads from all accessions were pooled and then mapped to the reference genome.


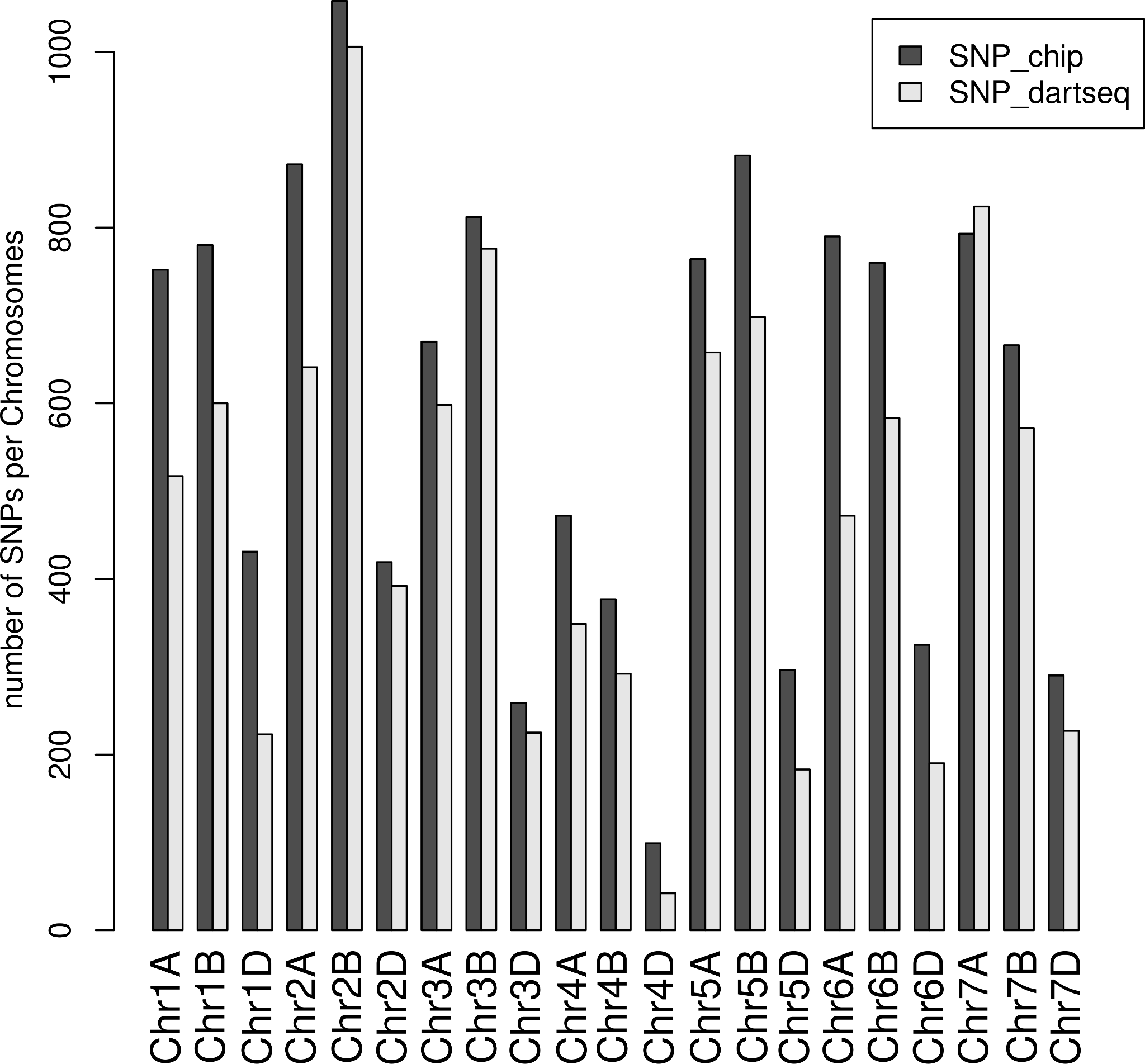


**Fig S6**: Barplot showing the number of SNPs per chromosome of the wheat genome for the SNP_chip matrix and the SNP matrix created from the DArTseq data. The dark bar corresponds to the SNPs chip dataset and the grey bar to the DArTseq dataset.


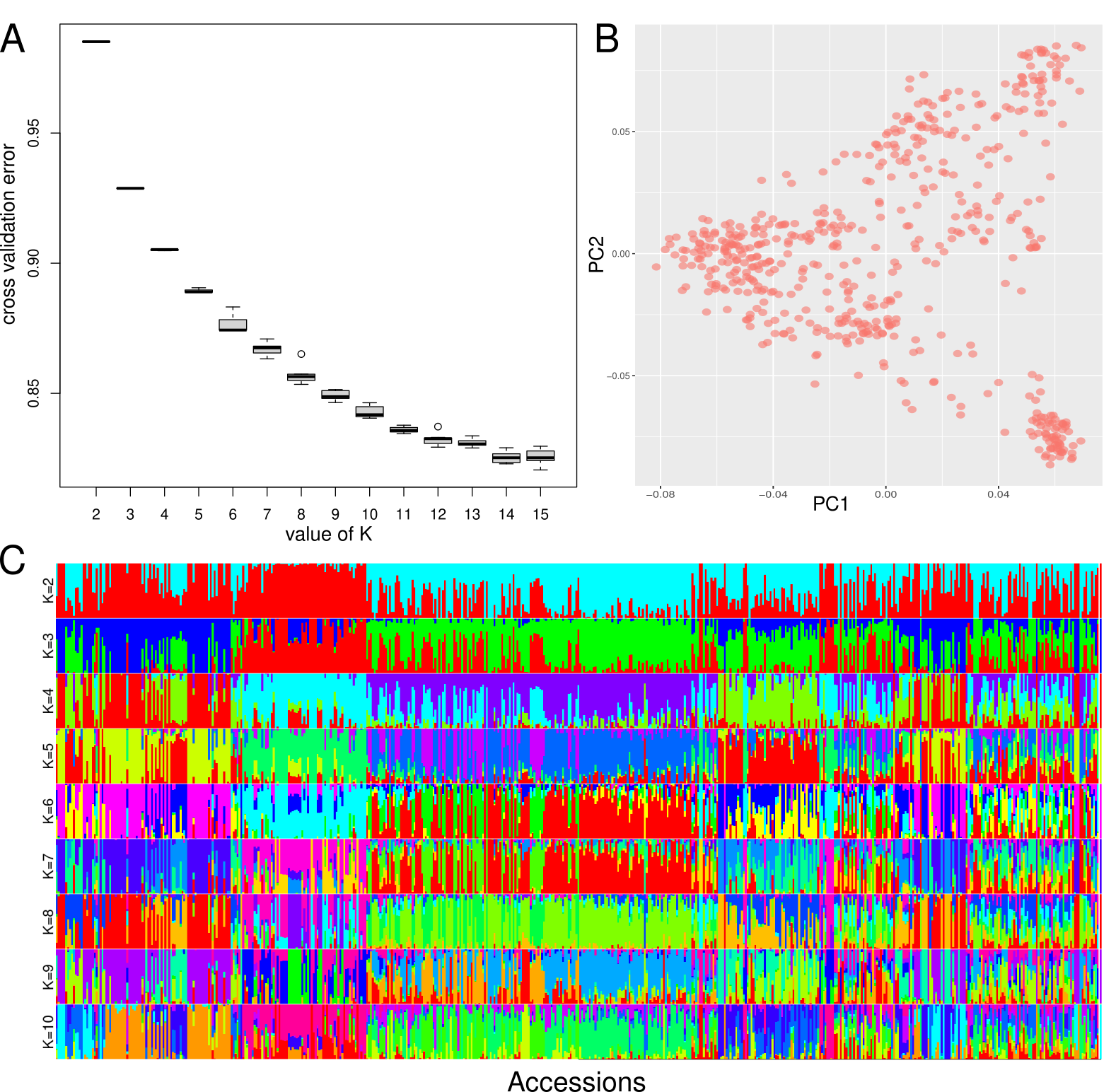


**Fig S7**: Population structure analysis of the Swiss wheat collection using ADMIXTURE. (A) Boxplot showing the variation (10 replicates) of cross-validation error (y-axis) for different values of K (x-axis). (B) PCA analysis of the 461 accessions based on the SNP matrix derived from the DArTseq sequencing. (C) Admixture plot of all 416 accessions for K values ranging from K=2 to K=10.


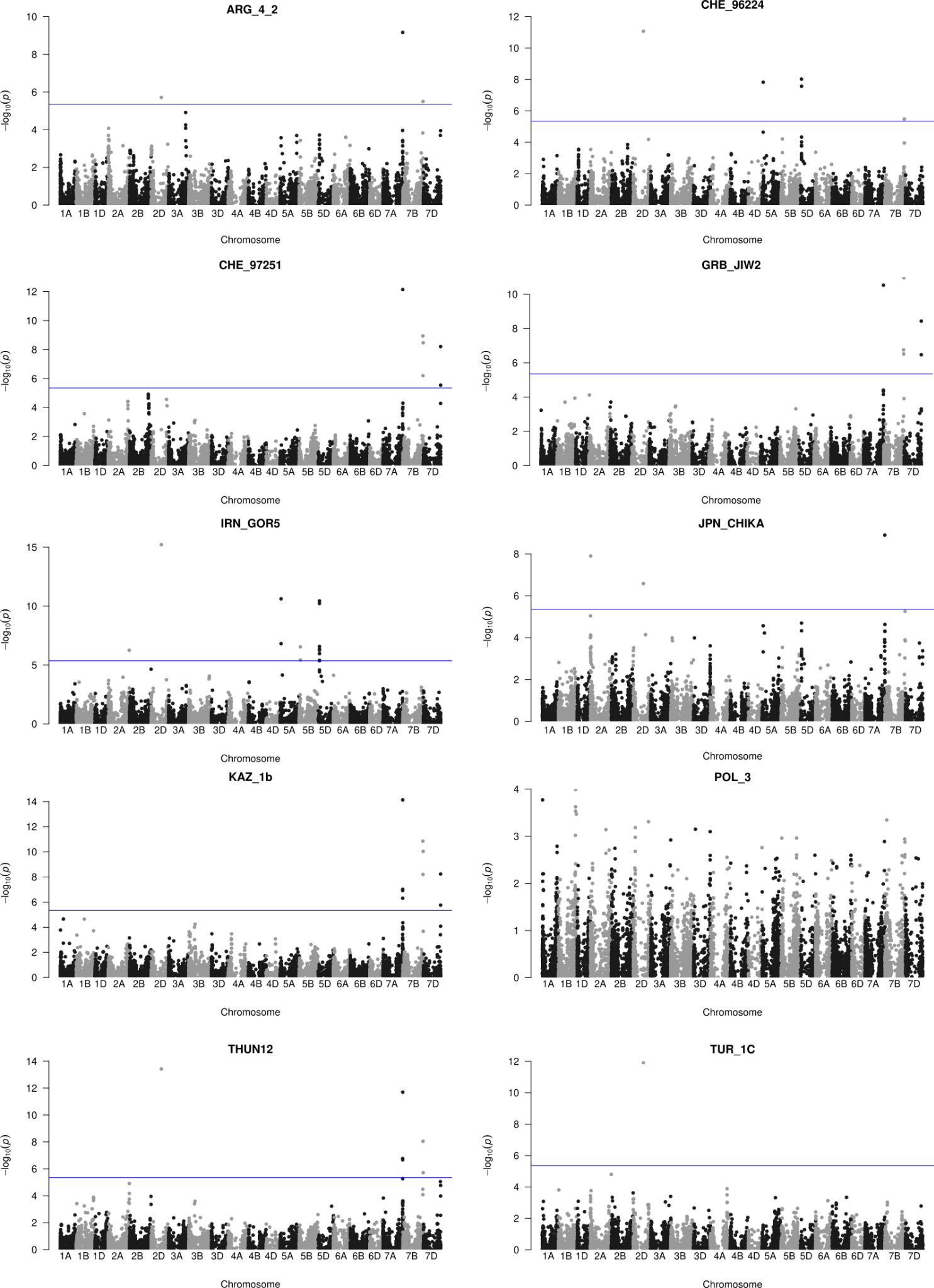


**Fig S8:** Manhattan plots using CS as reference genome. Each plot shows the GWAS of one of the 10 isolates used for phenotyping. The SNP matrix generated using an SNP chip has been used as genotype. The blue line represents the Bonferroni threshold. The scale is adapted to each plot.


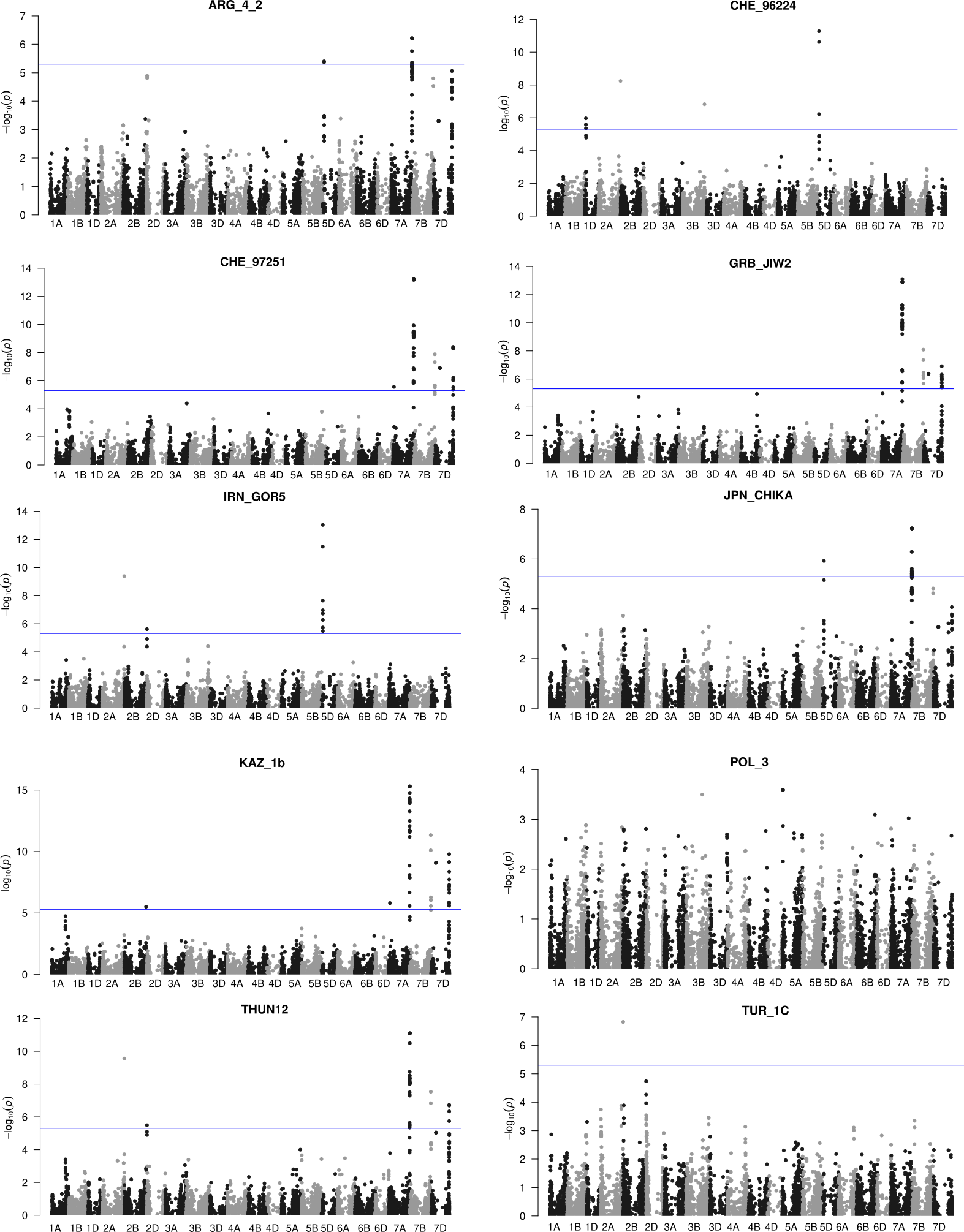


**Fig S9:** Manhattan plots using CS as reference genome. Each plot shows the GWAS of one of the 10 isolates used for phenotyping. The SNP matrix used as genotype was generated from the DArTseq mapped to CS. The blue line represents the Bonferroni threshold. The y-axis scale is adapted to each plot.


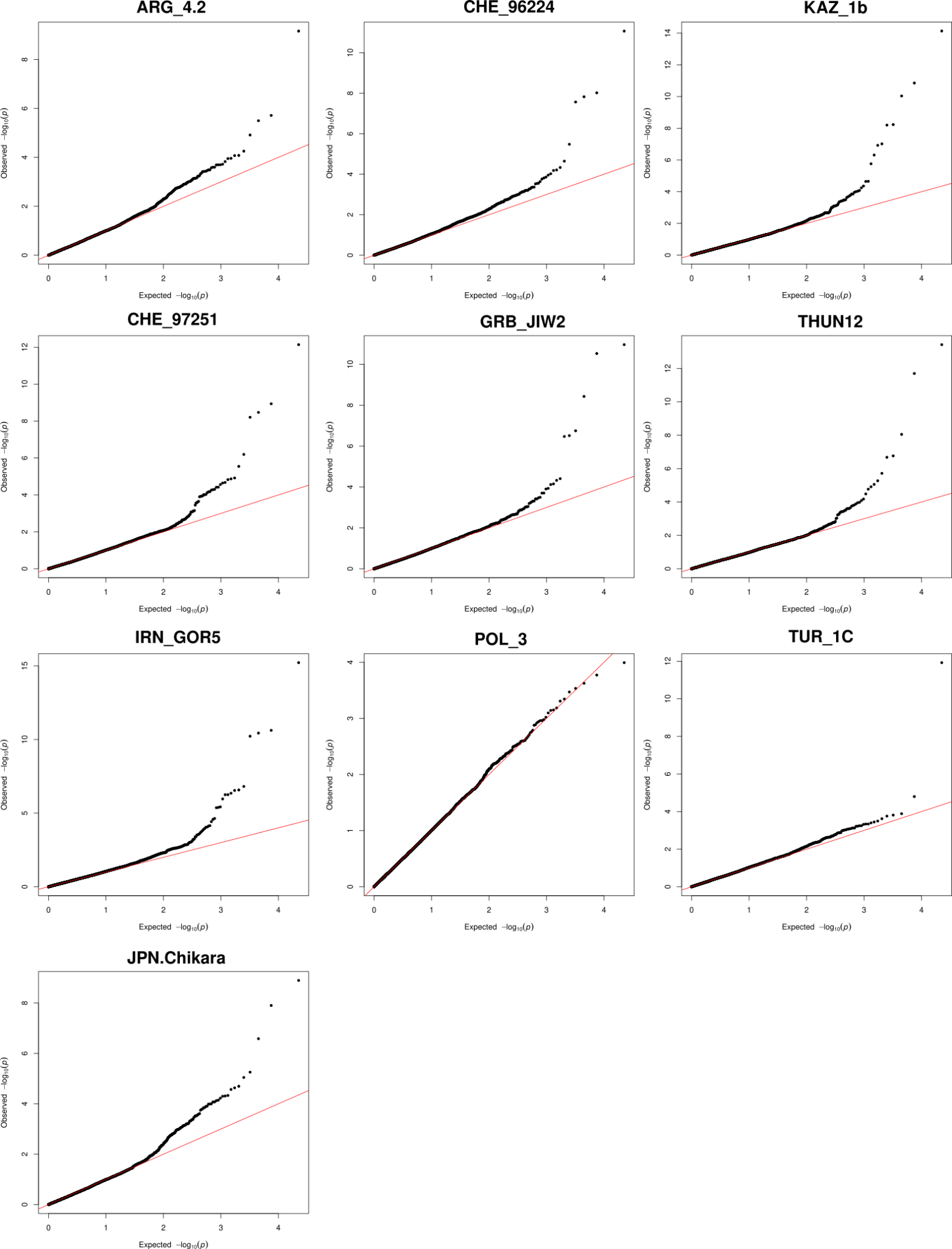


**Fig S10**: **QQplots for each of the *Bgt* isolates used as phenotypes in this study.** Plots are based on the GWAS results using the SNPs generated by the Illumina Infinium 15K wheat SNP array (TraitGenetics GmbH, Gatersleben, Germany) as genotype. In all cases, the expected and the observed distribution of P-value are well correlated, indicating that the GWAS model fits the data well.


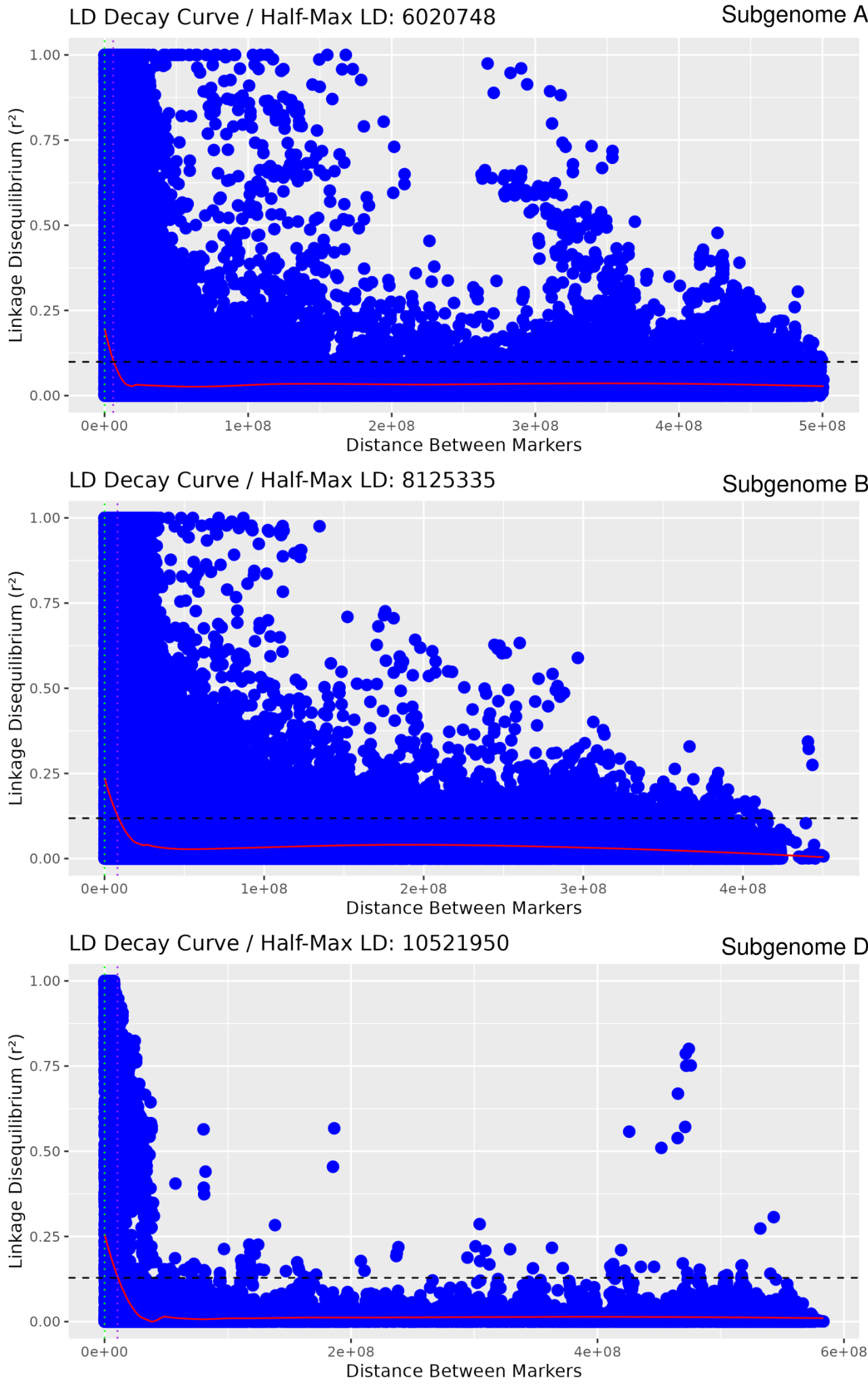


**Fig S11:** LD decay curves calculated for each of the subgenomes (A, B and D) of the hexaploid wheat genome. The number on top of each plot represents the distance (in base pairs) corresponding to half of the maximum LD.


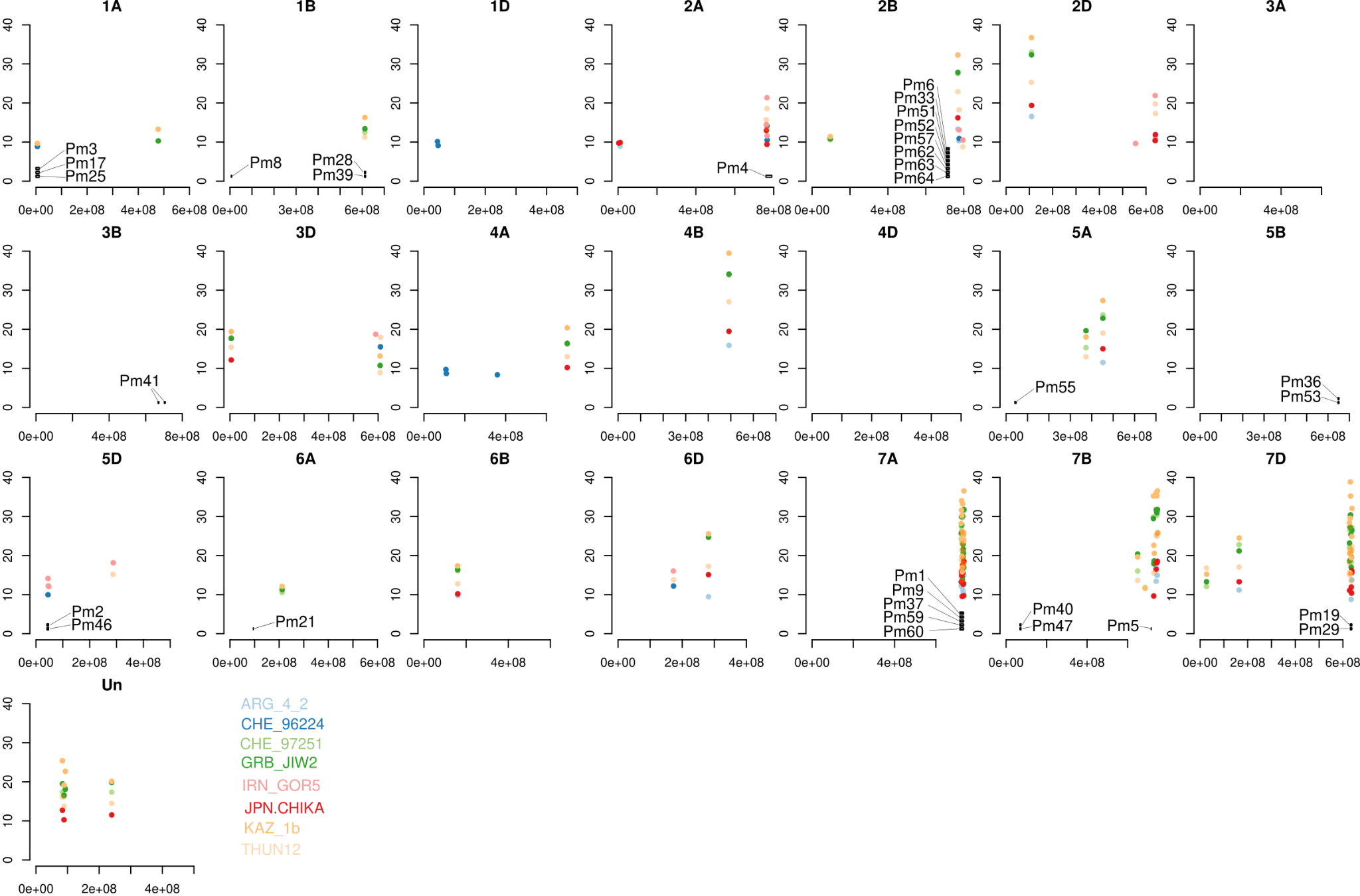


**Fig S12:** Manhattan plot from *k*-mer GWAS using the CS genome as a reference. *Pm* gene locations are inferred from blast and previous publications (**Additional file 2: Table S1 sheet 9**). Colors represent the different isolates.


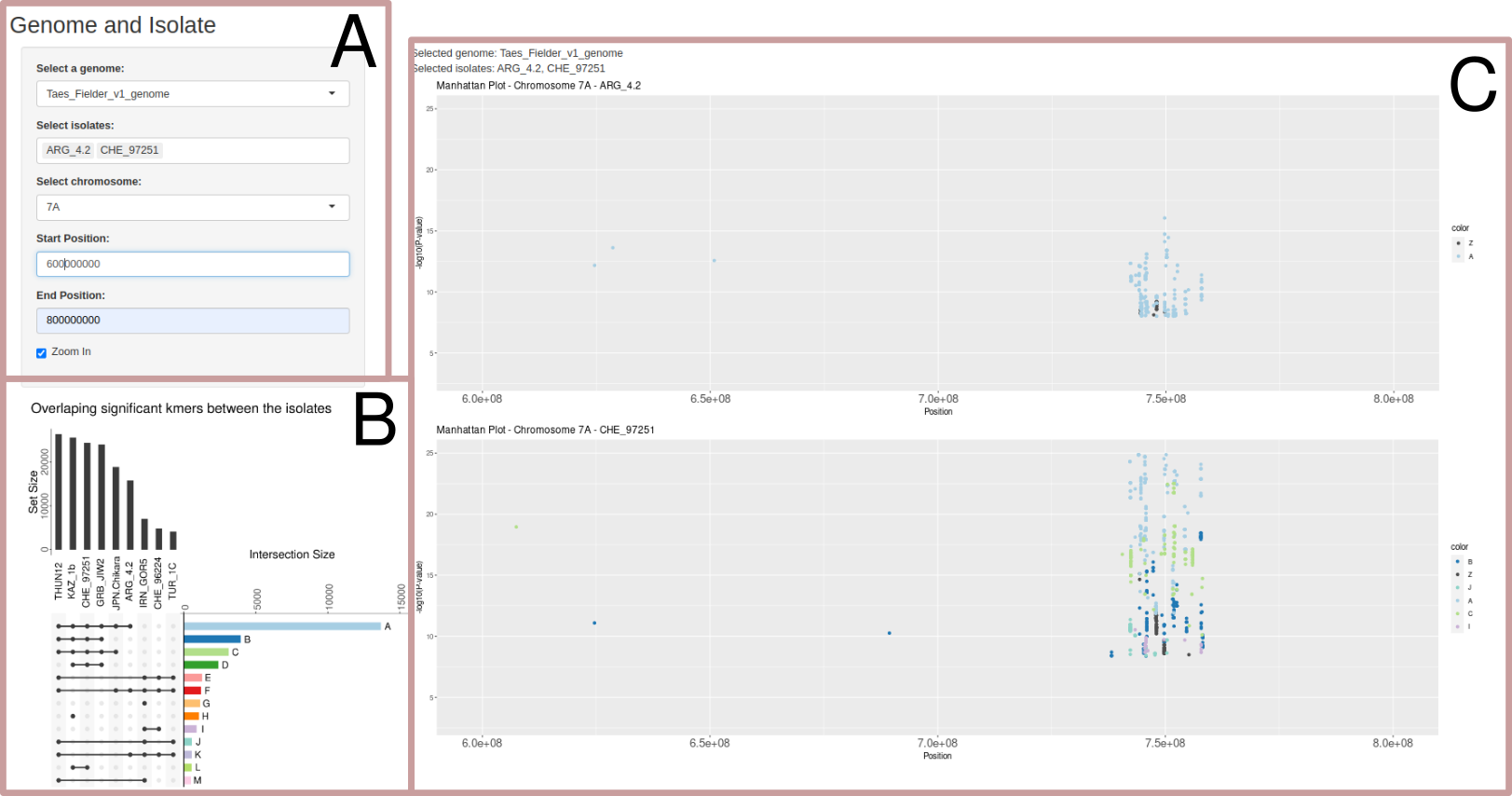


**Fig S13**: Screenshot of the Shiny app to explore the GWAS data. Panel A shows the selection of the genome of reference, the isolates to display, and the chromosome. Another option is to zoom in on a specific region by giving the start and the end of the region in bp. Panel B shows the upset plot as in Figure 4A. The different groups are colored as displayed on the GWAS plot (Panel C).


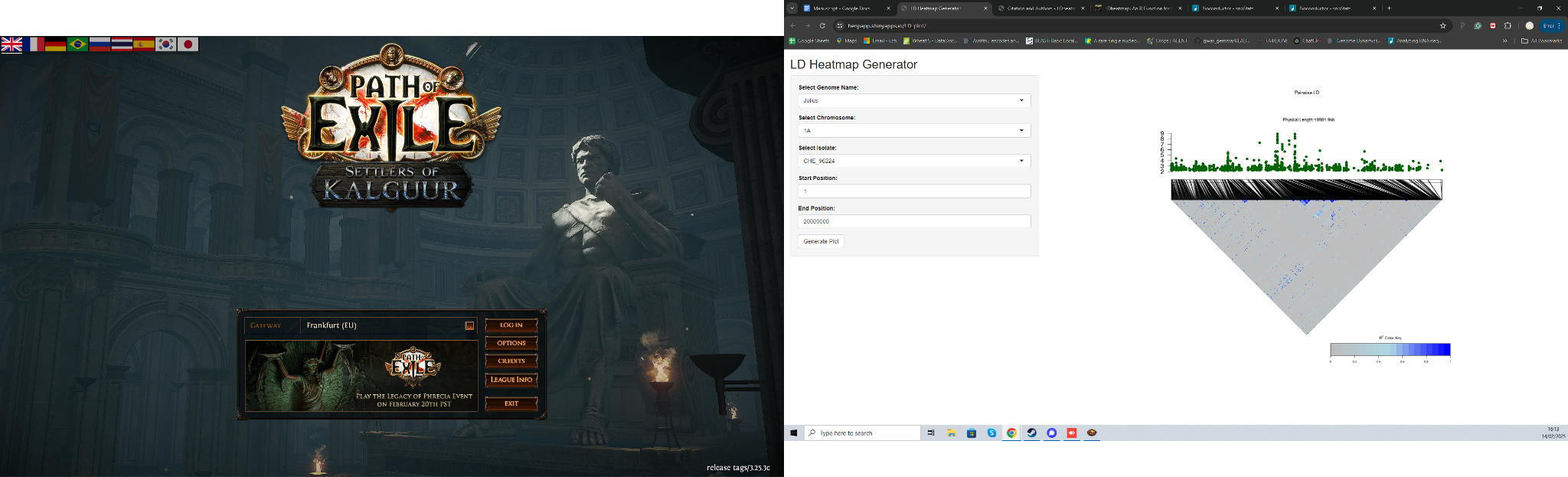


**Fig S14:** LD_plot Shiny App**.** The LD_plot Shiny app allows users to visualize linkage disequilibrium (LD) in a selected genomic region. Users can define the region of interest by specifying the genome, chromosome number, the isolate used as the phenotype for the GWAS, and the start and the end positions in base pairs (bp) as well. After entering these parameters, clicking the “Generate Plot” button will generate an LD plot on the right panel. The time required to generate the plot depends on the size of the selected regions and the number of markers present. For larger regions with many markers, the process may take several minutes.


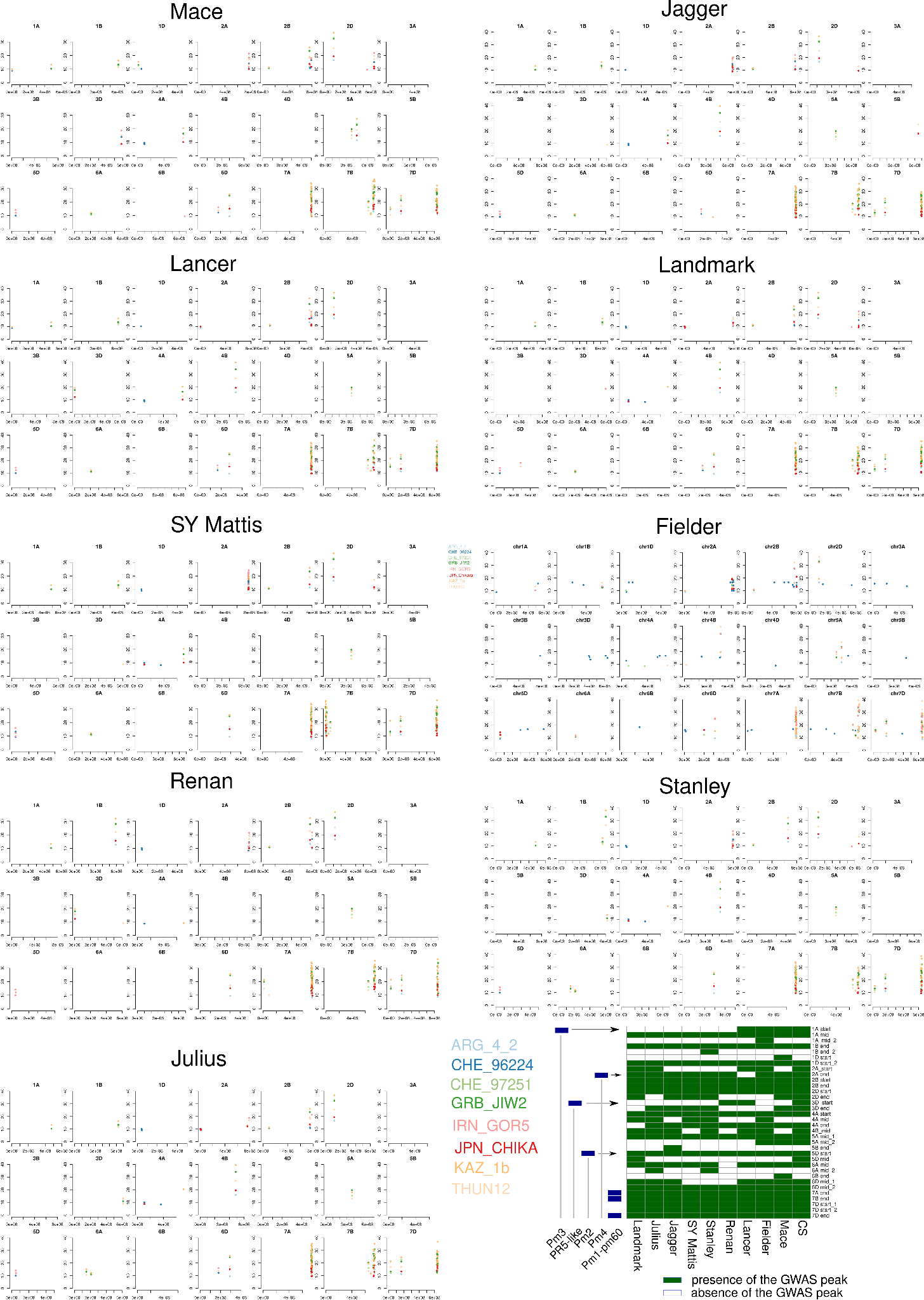


**Fig S15:** Manhattan plots of nine reference genomes where different colors represent associations for the various isolates. The positions of the *k-*mers represented have been filtered so that at least 10 *k-*mers are present per 1Mb regions as explained in the method and supplemental figure 23. A summary table describes the presence and absence of the main peaks based on their overall chromosomal positions.


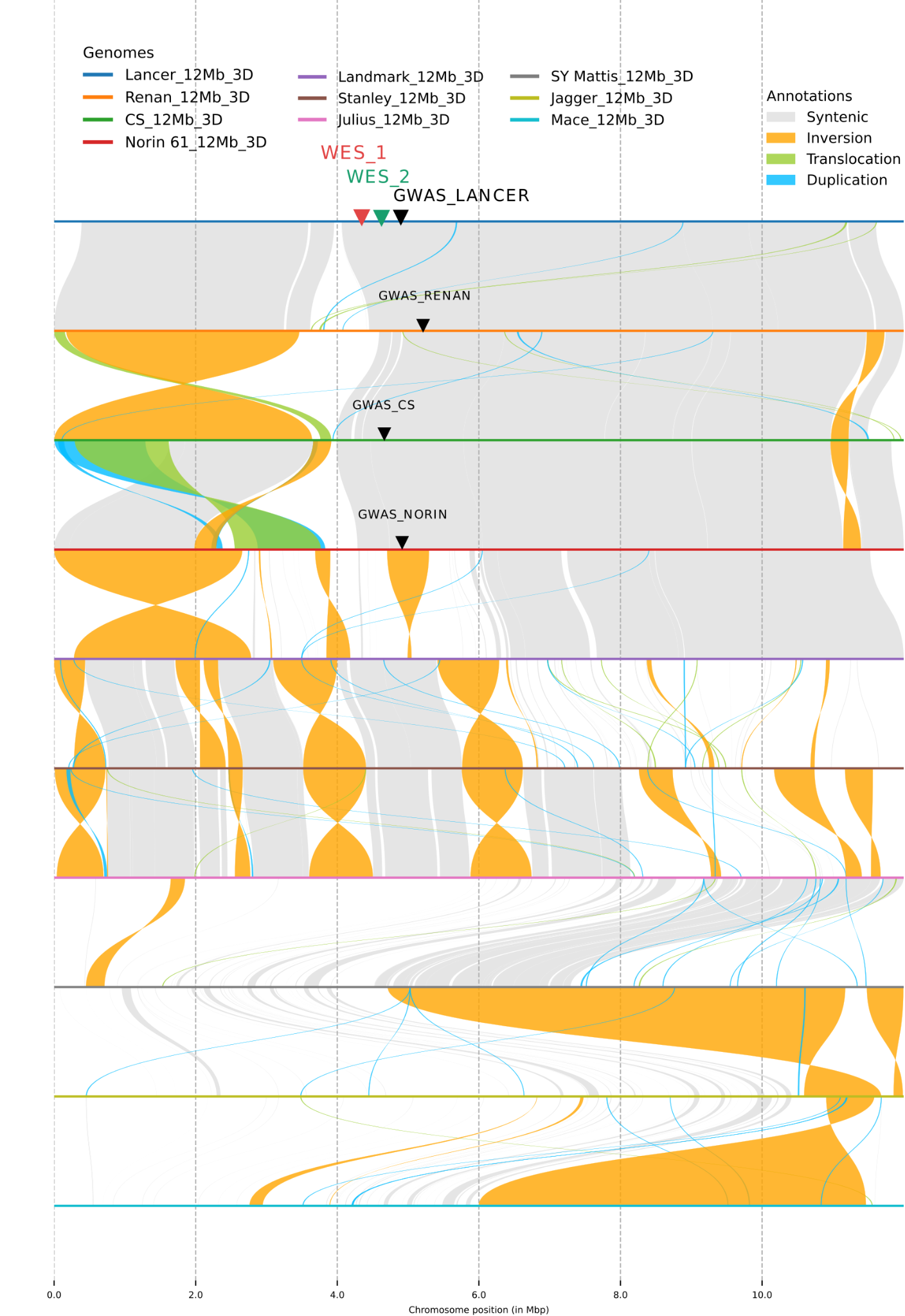


**Fig S16**: Genome alignment of the first 12Mb of chromosome 3D for all the genomes. WES 1 and 2 represent the position of the wax ester synthase candidate. Each section represents 2 Mb.


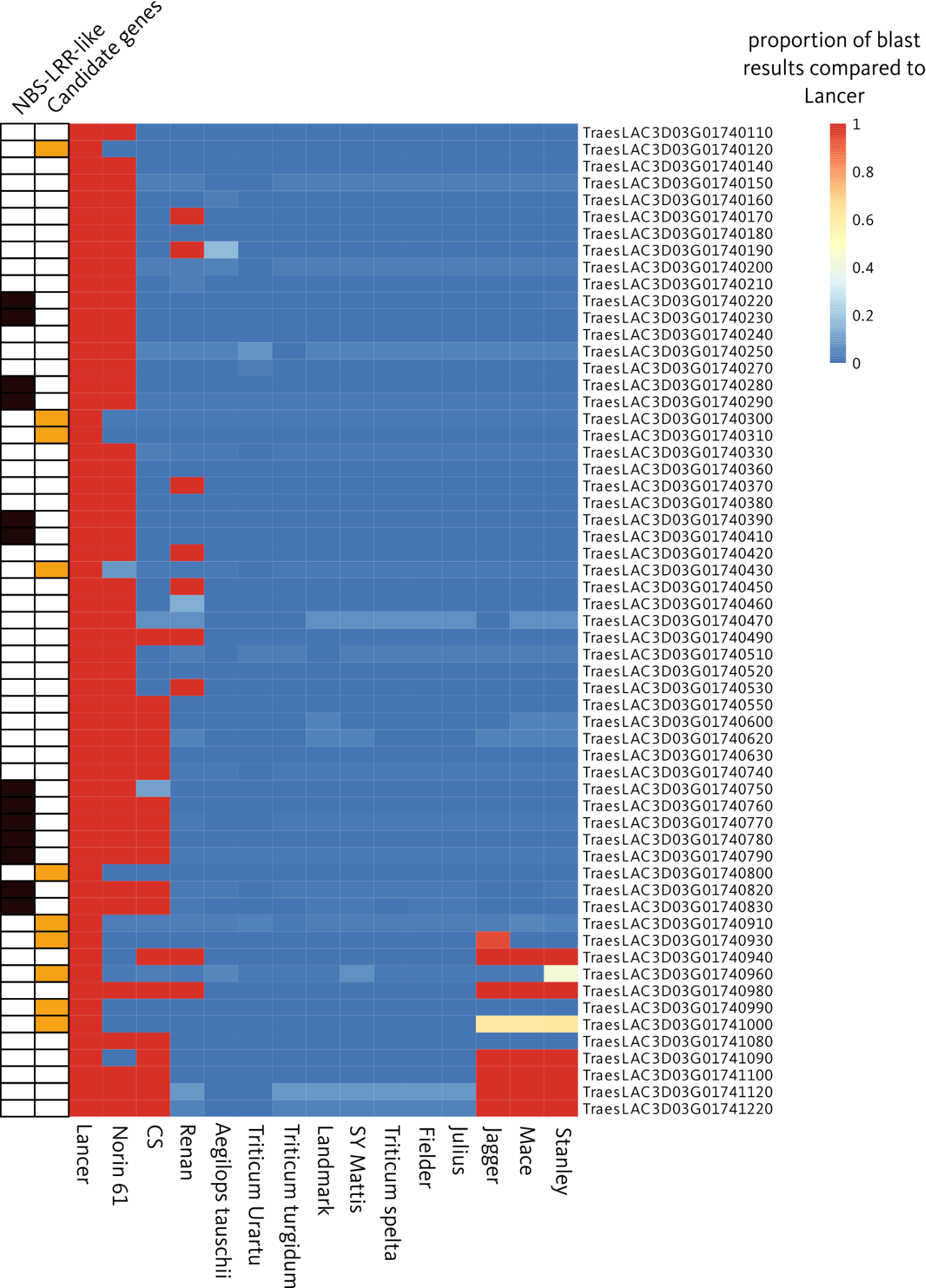


**Fig S17: Conservation of genes in the region of interest in the genome of Lancer compared to different wheat reference genomes.** A total of 59 genes, depicted as rectangles in the left of the heatmap, were extracted from the Lancer genome and blasted against other genomes. Black-and-white side rectangles indicate NBS-LRR-like genes. In the heatmap, the color scale represents the proportion of the gene length that perfectly matches the corresponding gene in Lancer, with red indicating a high degree of conservation (1.0) and blue indicating low or no conservation (0.0). The y-axis lists the genes identified in Lancer, while the x-axis represents different wheat lines or species. The 10 candidate genes private to Lancer representing candidate genes are highlighted in orange.


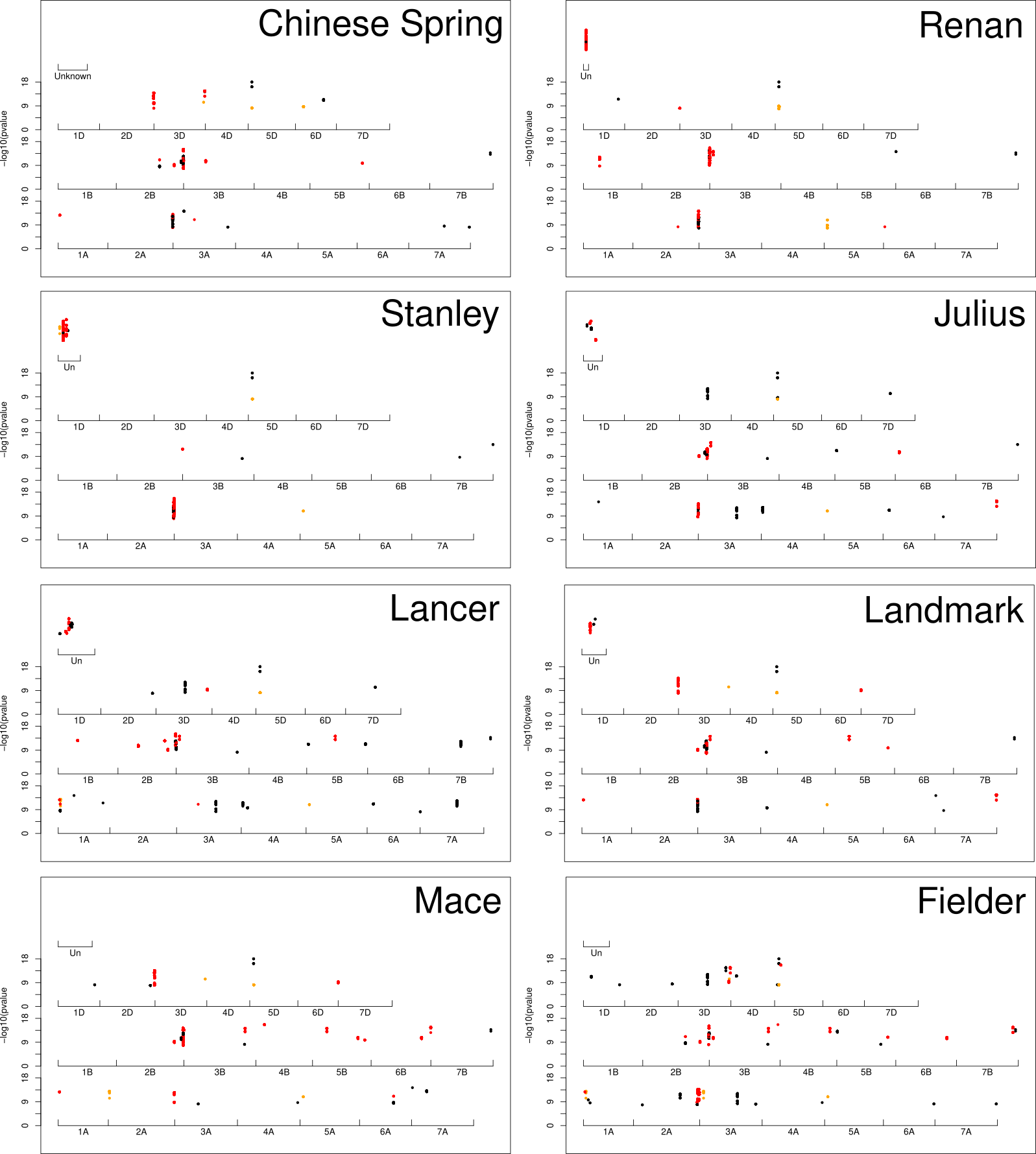


**Fig S18**: Manhattan plot of resistance-associated loci against the CHE_96224 isolate using eight different wheat genomes as a reference. Only the significantly associated *k*-mers are plotted. All the significant *k*-mers have been used and have been colored based on the result using the SY Mattis genome as presented in Figure 1F. Red *k*-mer is found to be associated with the region around *Pm4,* and orange with the region around *Pm2.* Black was not found in any of those two regions using the SY Mattis genome as a reference.


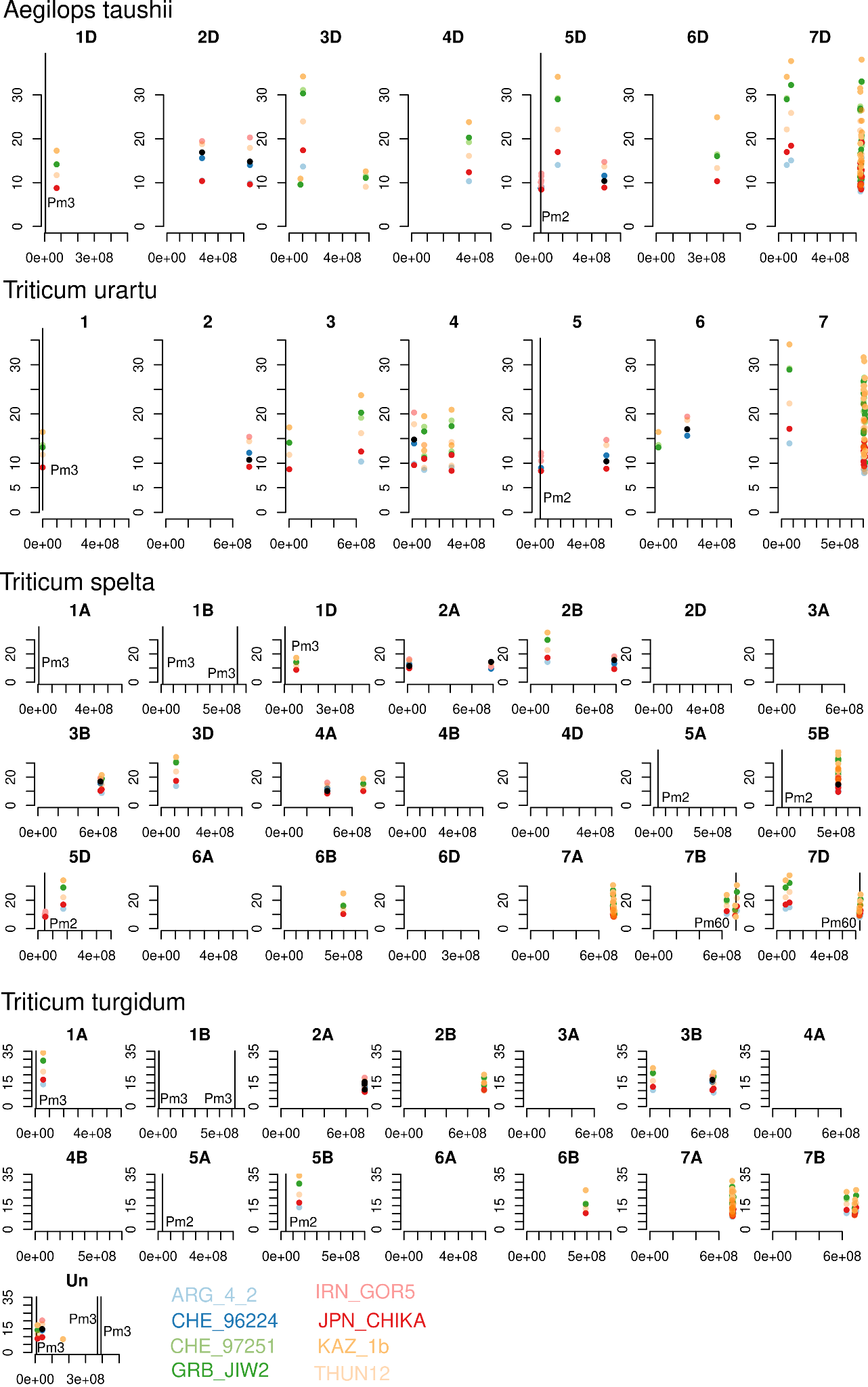


**Fig S19:** Manhattan plot using the progenitor genomes as reference. Colors represent powdery mildew isolates from which the significant *k-*mers have been detected, as depicted in the figure. Additionally, the position of *Pm* genes from blasting gene sequences to the genomes is displayed.


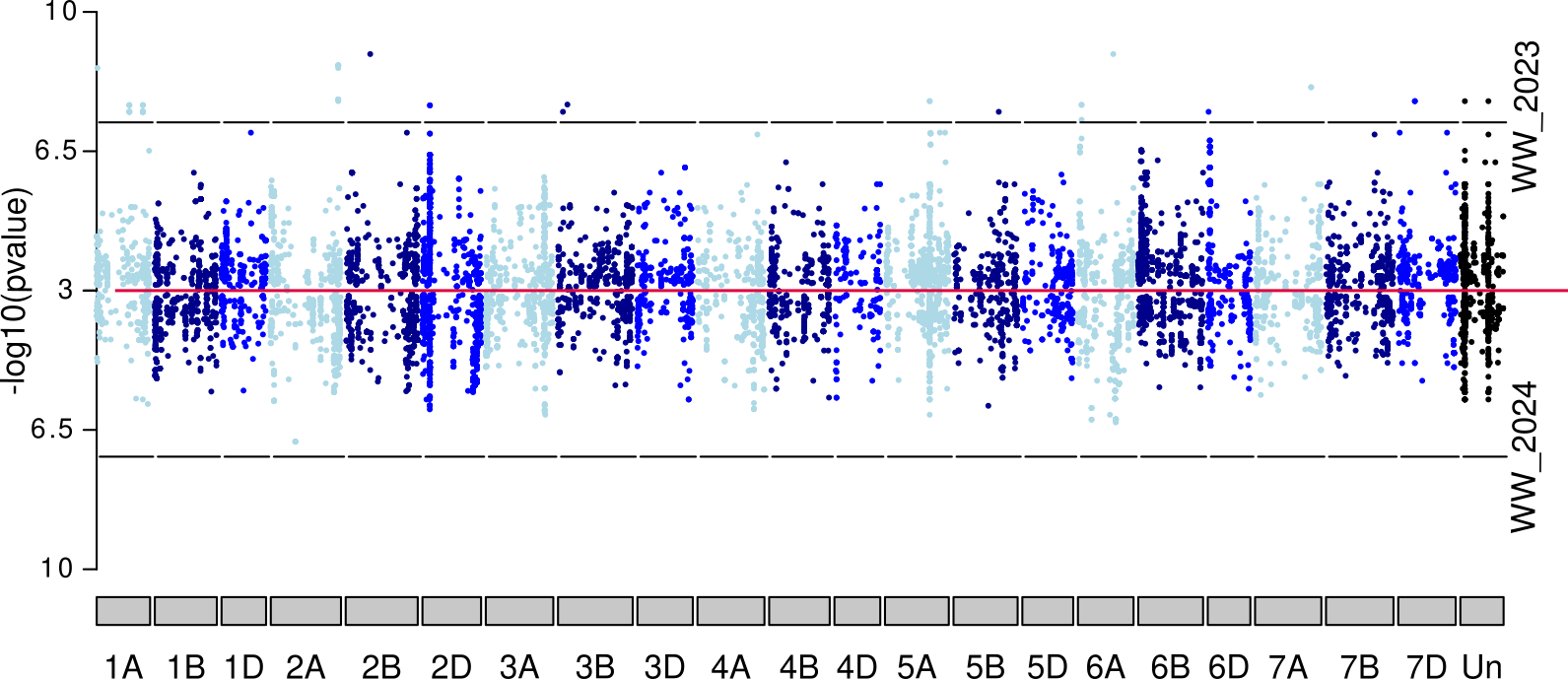


**Fig S20**: Manhattan plot comparing field resistance for winter wheat from trials in 2023 and 2024. The -log10(p-values) have been plotted from 3 onward. The red line separates the two Manhattan plots. The black dotted line represents the Bonferroni correction threshold. This plot is based on the CS reference genome.


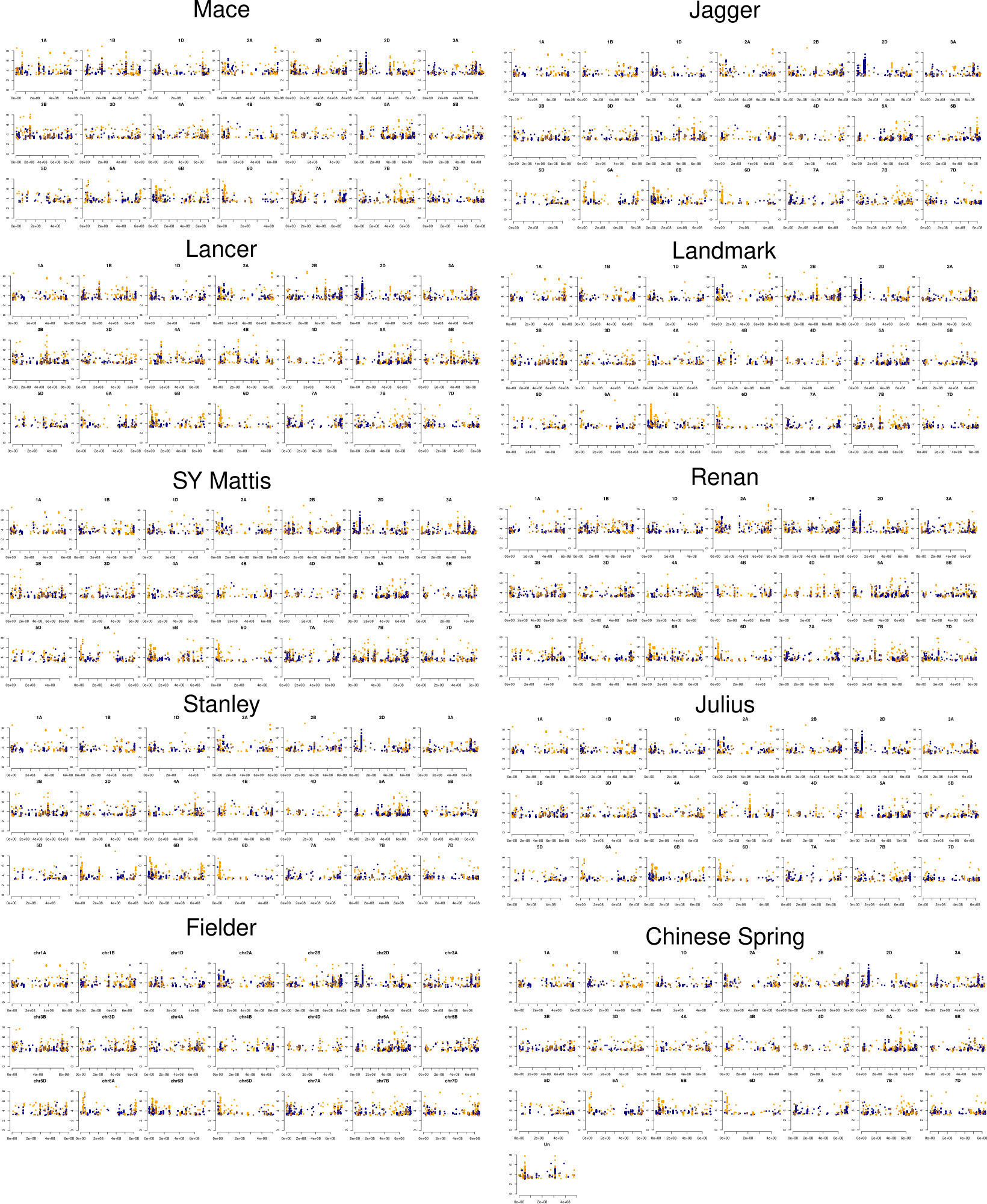


**Fig S21**: Manhattan plot for field resistance to powdery mildew for the winter wheat field experiment 2023. The 10 reference genomes used in this study are represented. The colors represent the allele frequency of the *k*-mers with orange for extreme frequencies (close to 0 or 1) and blue for frequencies around 0.5


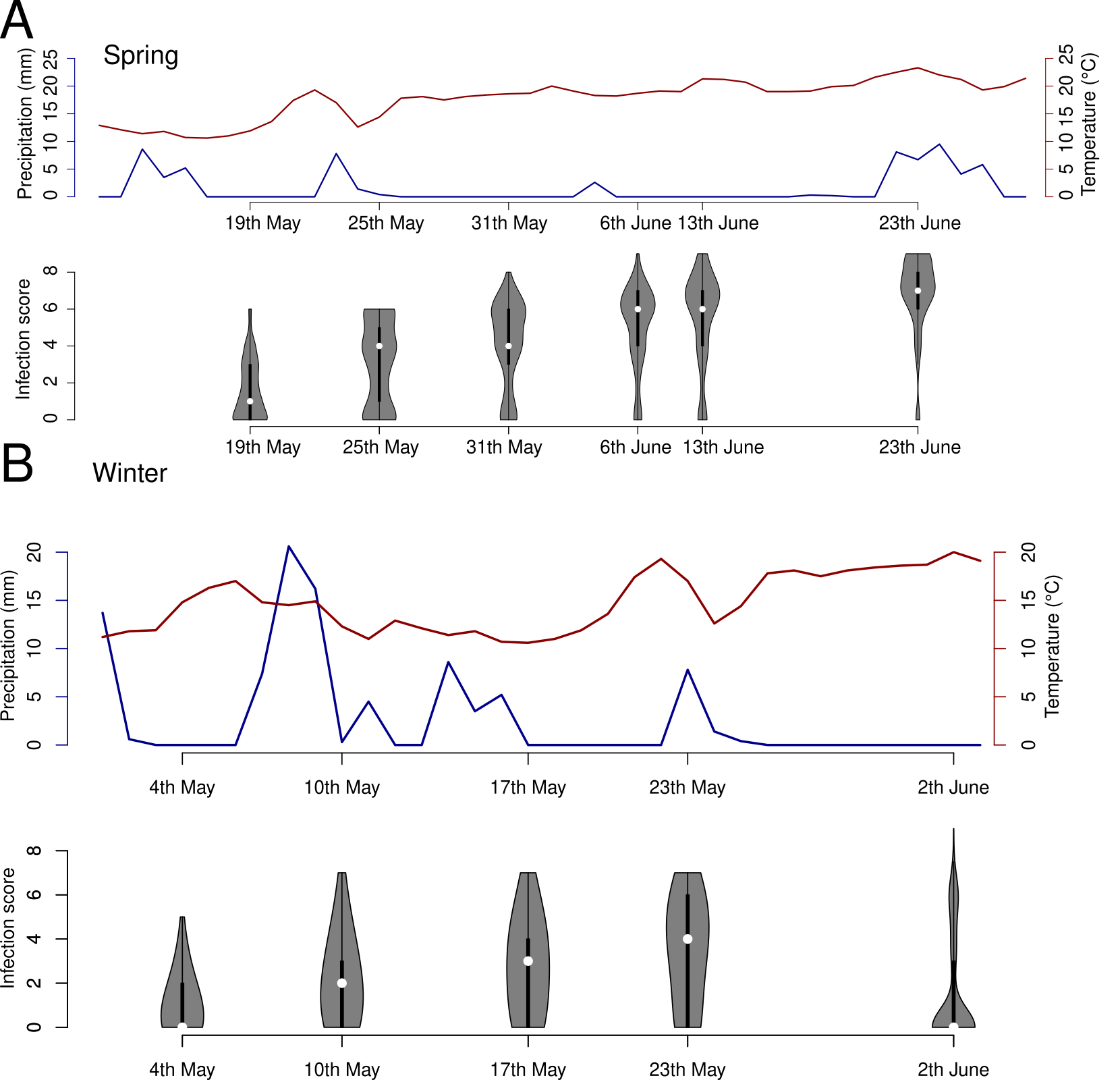


**Fig S22**: Phenotype distribution of the field data 2023 separated based on the phenotyping dates with the corresponding temperature and precipitation for Spring (A) and Winter (B) wheat trial


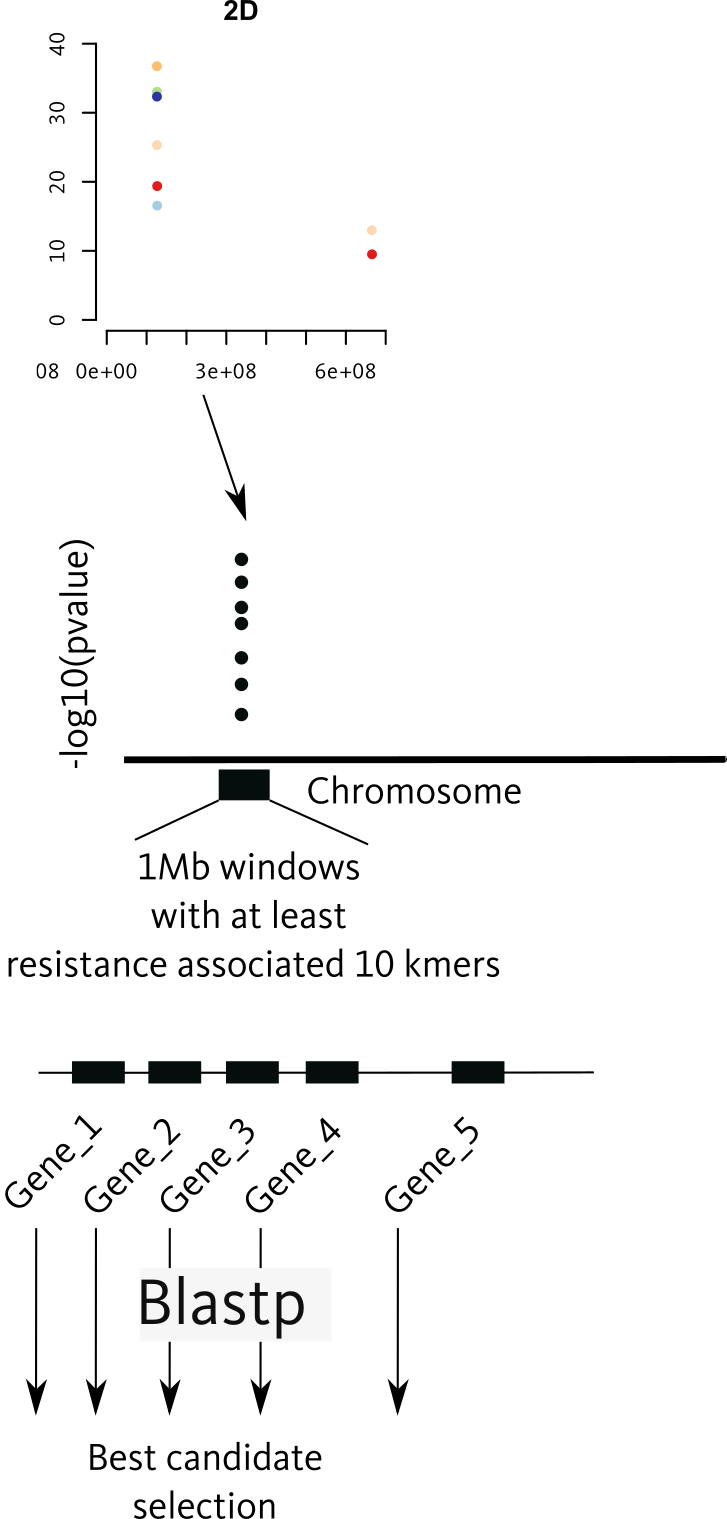


**Fig S23**: Candidate selection approach: For each of the associated regions detected, a region of 1 Mb around the most significantly associated SNPs was extracted, and all the protein sequences of the annotated genes within the region were extracted. The protein sequences of interest were then blasted to the NCBI database to assign a function to each gene (see Methods).
